# Sterol Endoperoxides and Their Antileishmanial Effects: Influence on Viability, Oxygen Metabolism and Sterol Synthesis

**DOI:** 10.3390/molecules31060979

**Published:** 2026-03-14

**Authors:** Deblina Sarkar, Azra Aleta, Moris Ahmetašević, Mira Tosin, Laura Machin, Elisabeth Schrödl, Markus Bacher, Thomas Rosenau, Lianet Monzote, Katrin Staniek, Mitali Chatterjee, Lars Gille

**Affiliations:** 1 Pharmacology and Toxicology, Department of Biological Sciences and Pathobiology, University of Veterinary Medicine, 1210 Vienna, Austria; deblinasarkar09@gmail.com (D.S.); azraaleta@icloud.com (A.A.); morisahm@gmail.com (M.A.); mira.tosin@yahoo.com (M.T.); laura@ifal.uh.cu (L.M.); elisabethschroedl@gmx.at (E.S.); katrin.staniek@vetmeduni.ac.at (K.S.); 2 Department of Pharmacology, Institute of Postgraduate Medical Education and Research, Kolkata 700 020, India; ilatimc@gmail.com; 3 Pharmacy Department, Institute of Pharmacy and Food Sciences, University of Havana, Havana 13600, Cuba; 4 Institute of Chemistry of Renewable Resources, Department of Natural Sciences and Sustainable Resources, UFT Research Center, BOKU University, 3430 Tulln, Austria; markus.bacher@boku.ac.at (M.B.); thomas.rosenau@boku.ac.at (T.R.); 5 Parasitology Department, Institute of Tropical Medicine “Pedro Kouri”, Havana 17100, Cuba; monzote@ipk.sld.cu

**Keywords:** *Leishmania*, macrophages, sterol endoperoxides, mitochondria, viability, 5-dehydroepisterol

## Abstract

Leishmaniasis is a global health issue, especially in tropical and subtropical areas, with treatment challenges due to the development of resistance to current drugs. This has prompted the search for new antileishmanial compounds. Endoperoxides, due to parasites’ reliance on external iron and susceptibility to oxidative stress, are promising antileishmanial compounds. This study evaluated two sterol endoperoxides—ergosterol endoperoxide (ErgoEP) and dehydrocholesterol endoperoxide (DHCholEP)—for their antileishmanial activity and mechanism in vitro. Cell viability assays with *Leishmania donovani* and *Leishmania tarentolae* promastigotes showed IC_50_ values in the low micromolar range (from 2.0 to 4.5 µM, respectively) with low toxicity to murine and J774A.1 macrophages. Electron paramagnetic resonance spectroscopy confirmed radical generation in the presence of low-molecular-weight iron compounds. However, this did not trigger the antileishmanial effect, as neither N-acetylcysteine nor pyridoxal isonicotinoyl hydrazone altered activity. Mitochondrial function(s) and superoxide production in *Leishmania* remained unaffected. Both endoperoxides significantly inhibited synthesis of 5-dehydroepisterol, the major sterol in *Leishmania tarentolae*, suggesting targeting of the sterol biosynthesis pathway. Their limited toxicity to mammalian macrophages makes ergosterol and dehydrocholesterol endoperoxides promising candidates for future antileishmanial drug development.

## 1. Introduction

*Leishmania* are intracellular protozoan parasites responsible for a group of tropical diseases known as leishmaniasis [1]. This disease affects people in tropical and subtropical regions reaching from Africa and Asia to North and South America as well as Europe [2,3,4]. With its impact spanning 98 countries and affecting at least two million individuals annually, and posing a threat to 350 million, leishmaniasis is recognized as a neglected tropical disease by the World Health Organization and is therefore considered a global health issue [1].

The life cycle of *Leishmania* parasites consists of two main stages, promastigotes in the sandfly vector and amastigotes in mammalian host macrophages [1]. In the promastigote stage, *Leishmania* are flagellated and motile, allowing attachment to the sandfly’s intestines, while the amastigote form in the mammalian host is non-flagellated [5].

Preventative strategies, such as early diagnosis and treatment and control of the sandfly vector and the animal reservoirs along with disease surveillance, aim to reduce transmission and spread of the disease, as well as increase the chance of improved recovery for individuals already infected. Nevertheless, chemotherapy for leishmaniasis remains the most important measure to control this disease. Currently available drugs are limited in number and have limitations, such as mammalian toxicity, high production cost, administration difficulties and decreasing efficacy due to development of resistance [6,7]. This situation obligates the scientific community to search for new active antileishmanial compounds that are effective and easily accessible with minimal mammalian toxicity.

Considering the high iron demand of intracellular amastigotes and promastigotes, the strategy of targeting these parasites by oxidizing agents has been extrapolated from antimalarial artemisinins, which are endoperoxides (EPs). A characteristic structural feature of EPs is the peroxide group (O–O), which connects in a cyclical manner to carbon atoms of the residual molecule [8,9,10]. EPs are widely spread in nature and exist in organisms as signaling intermediates (mammals) or secondary metabolites (plants) [11]. Well-known EP intermediates in prostanoid synthesis in mammals are prostaglandins PGG_2_ and PGH_2_, which are formed by cyclooxygenases from arachidonic acid [12]. Many plant-derived EPs are defense molecules known to be effective against bacteria, fungi and protozoa, including parasites such as *Trypanosoma cruzi* [10]. Moreover, EPs exhibit anticancer activity in colorectal cancer, hepatocellular carcinoma and leukemias, e.g., ergosterol EP [13]. EPs are known for their rapid reaction with biologically relevant transition metals, such as Fe^2+^ and Cu^+^ [14]. We have previously demonstrated the antileishmanial activity of natural EPs, such as ascaridole from *Dysphania* (*Chenopodium*) *ambrosioides* (L.) Mosyakin & Clemants [15] and artemisinin from *Artemisia annua* [16], and artificial EPs, such as anthracene EPs [17]. For the activation of those Eps, the labile iron pool and heme compounds in *Leishmania* play an essential role and thereby simultaneously interfere in the iron supply of *Leishmania* necessary for their proliferation [18].

Therefore, it appeared of high interest to explore a further class of EPs for their antileishmanial action and mechanism, namely sterol-derived EPs. Lipids are a crucial element in the biology of protozoans and mammalian cells, while a major distinction between *Leishmania* and mammalian cells is the sterol composition of their membranes [19]. While for mammalian cells cholesterol-derived molecules dominate, *Leishmania* parasites rely on ergosterol and ergosterol-like sterols similar to fungi [19]. These sterols in *Leishmania* are involved in many cellular processes, such as energy production, membrane organization along with host–parasite interactions and even development of resistance [20,21]. The nature and occurrence of sterol oxidation products is profoundly different from one to another. Oxidation of cholesterol (Chol) requires strongly oxidizing agents and is of pathophysiological importance in mammalian degenerative diseases [22]. In contrast, ergosterol (Ergo) can more easily undergo oxidation, leading to various compounds including ergosterol endoperoxide (ErgoEP) [23]. ErgoEP is a naturally occurring secondary plant metabolite found especially in mushrooms [10]. It was isolated in the past from different edible mushrooms [24] and its possible pharmacological applications against bacteria, viruses and cancer cells were demonstrated [10,13]. Although the activity of ErgoEP in *Leishmania* has been reported [25], the efficiency and mechanism of sterol EPs against *Leishmania* has not yet been delineated.

Therefore, we synthesized ErgoEP from Ergo and dehydrocholesterol endoperoxide (DHCholEP) from 7-dehydrocholesterol (DHChol) and studied their antileishmanial effects, establishing their mechanism on *Leishmania donovani* (Ld) and *Leishmania tarentolae* (Lt) using in vitro models.

## 2. Results

### 2.1. Synthesis of Sterol EPs

**Figure 1:**
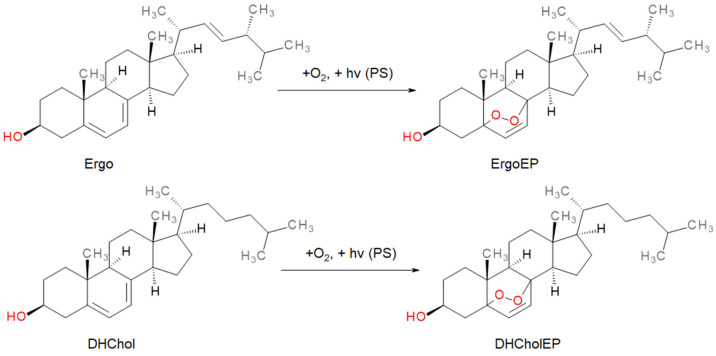
Synthesis of sterol EPs. Ergosterol (Ergo) and dehydrocholesterol (DHChol) were subjected to visible light irradiation (hv) in the presence of the photosensitizer (PS) methylene blue and oxygen, yielding corresponding endoperoxides ErgoEP and DHCholEP, respectively.

Both sterol EPs (ErgoEP and DHCholEP) were synthesized from their parent sterols, Ergo and DHChol, using a photochemical method with methylene blue as photosensitizer (Figure 1). The products were shown to contain >99% EPs and minor traces of isomers with an additional double bond (DHErgoEP, TDHCholEP).

### 2.2. Chemical Reactivity of Sterols and Their EPs

We studied the reaction of EPs and parent sterols with Fe^2+^ using xylenol orange (XO) as detecting agent for Fe^3+^ by complex formation (Table 1).

The sterol EPs (ErgoEP, DHCholEP) clearly exhibited high reactivity with Fe^2+^ in this test system. Additionally, the parent sterols (Ergo, DHChol) also showed in this chemical solvent system a rapid formation of Fe^3+^. In contrast to these triene sterols (with conjugated double bond), Chol did not trigger significant Fe^3+^ formation at all, suggesting that the diene system in Ergo and DHChol may be prone to prooxidative reactions in the presence of O_2_.

**Figure 2:**
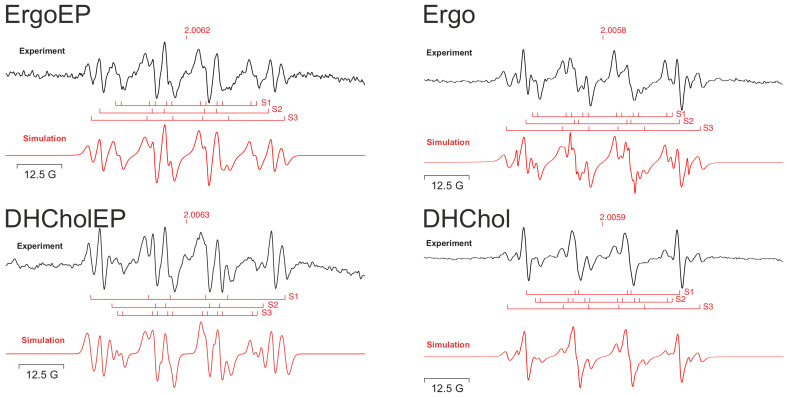
Typical EPR spectra of DMPO spin adducts obtained by the reaction of sterol EPs and sterols in the presence of Fe^2+^. Sterols/sterol EPs (900 µM) and (NH_4_)_2_Fe(SO_4_)_2_ (900 µM) were mixed in the presence of 20 mM DMPO in ACN/H_2_O (1:1 *v*/*v*). Experimental spectra are shown in black and simulated spectra in red. Splitting patterns of the three most abundant spin adducts for each compound are given below the experimental spectra (numbering according to Table 2). The g-values for the H0 are marked above experimental spectra.

The formation of radicals from sterol EPs and sterols was studied by electron paramagnetic resonance (EPR) spin trapping using 5,5-dimethyl-1-pyrroline-N-oxide (DMPO) as spin trap compound and Fe^2+^ (aquo complexes in the high-spin state arising from dissolution of (NH_4_)_2_Fe(SO_4_)_2_) (Figure 2). Not only the sterol EPs but also the sterols themselves yielded intense EPR spectra in the presence of Fe^2+^ (black lines). The obtained spectral patterns were simulated by the WINSIM 2002 software and are displayed as red lines below experimental spectra (Figure 2), showing an acceptable reproduction of experimental spectra. The parameters used for simulation are summarized in Table 2. Possible assignments were made according to [26].

The obtained simulation parameters suggest for all studied compounds the generation of both C-centered and O-centered radicals, which is compatible with one-electron reductive cleavage of both C–O–O–C and C–O–O–H groups. Nevertheless, spectral patterns and simulation parameters for corresponding pairs of sterol EP and parent sterol are different and suggest different peroxides to act as reactants for Fe^2+^. For sterol EPs, various cleavage products of one-electron reduction were expected as in the case of other EPs, such as ascaridole or artemisinin (Art). However, parent sterols may also give rise to intermediate formation of non-cyclic peroxides in the presence of O_2_ and Fe^2+^ in this system, explaining the spin adducts observed in the cases of these sterols (Table 2) and the rapid formation of Fe^3+^ in the XO assay (Table 1).

### 2.3. Influence of Sterol EPs on Viability

#### 2.3.1. LdP and MPM

**Figure 3:**
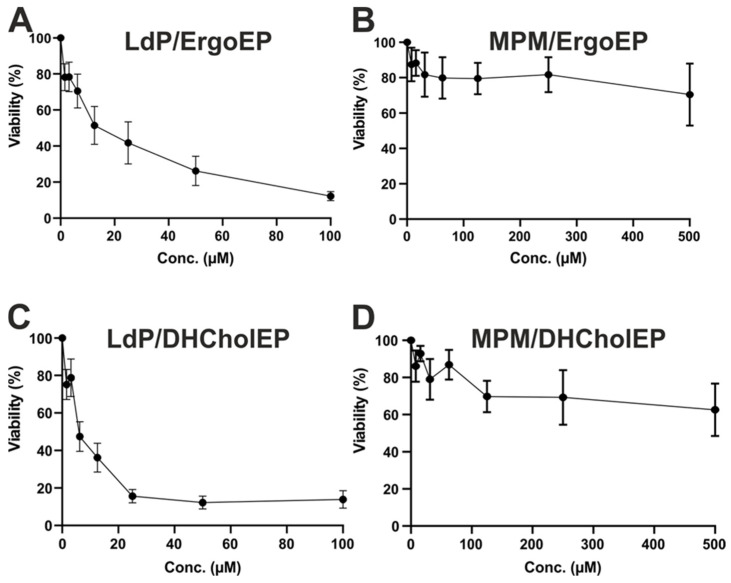
Antileishmanial efficacy of ErgoEP and DHCholEP against *Leishmania donovani* promastigotes (LdP) and cytotoxicity in mouse peritoneal macrophages (MPM). LdP (1 × 10^5^/200 µL/well) were incubated with (**A**) ErgoEP and (**C**) DHCholEP (0–100 µM) for 48 h at 24 °C. MPM were incubated with (**B**) ErgoEP and (**D**) DHCholEP (0–500 µM) for 48 h at 37 °C. Cell viability was measured by the MTS–PMS assay, and data are expressed as the mean ± SEM of at least three experiments in duplicates.

The anti-promastigote efficacy of ErgoEP and DHCholEP was initially studied in *L. donovani* promastigotes (LdP) cultured in M199 medium. The half-maximal inhibitory concentration (IC_50_) values of ErgoEP and DHCholEP were 18.3 ± 7.4 µM and 8.3 ± 2.1 µM, respectively (Figure 3A,C). The IC_50_ values of ErgoEP and DHCholEP in murine peritoneal macrophages (MPM) were >500 µM (Figure 3B,D), indicating selectivity towards parasites (Table 3). The selectivity index was calculated from IC_50_ or the highest concentration tested in MPM divided by IC_50_ of LdP (Table 3).

**Figure 4:**
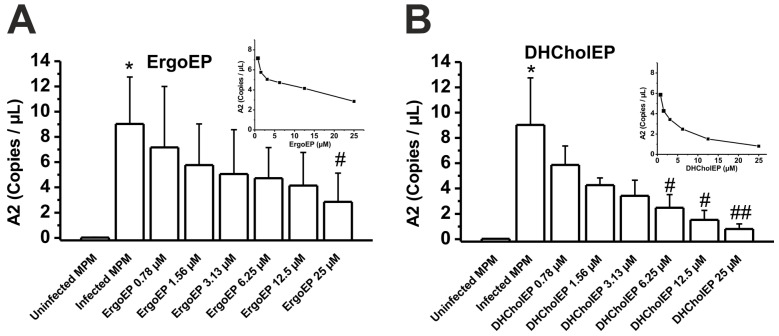
Evaluation of anti-amastigote efficacy of sterol EPs against *L. donovani*. Bar graphs show expression of *A2* gene in copies/μL (**A**,**B**) in uninfected and *Leishmania donovani*-infected mouse peritoneal macrophages (MPM). Infected macrophages were treated for 48 h with ErgoEP (0.78–25 μM; (**A**)) and DHCholEP (0.78–25 µM; (**B**)). In bar graphs, data are expressed as means ± SEM of at least three experiments in duplicates; * *p* < 0.0001 vs. uninfected MPM; ^#^ *p* < 0.05, ^##^ *p* < 0.01 vs. infected MPM. Insets show graphical extrapolations of IC_50_ values.

The activity of ErgoEP and DHCholEP against *Leishmania donovani* amastigotes (LdA) was quantified by ddPCR using the amastigote-specific *A2* gene in *L. donovani*-infected peritoneal macrophages (Figure S2A,B). While in uninfected MPM almost no *A2* gene was detected (0.02 ± 0.01 copies/μL), in infected MPM, 9.02 ± 1.67 copies/μL were observed. The incubation of infected MPM with ErgoEP concentration-dependently decreased the expression of the amastigote-specific gene, with an extrapolated IC_50_ value of 8.1 µM ErgoEP (inset, Figure 4A). Even more pronounced, DHCholEP progressively decreased the amastigote-specific gene in infected MPM, with an extrapolated IC_50_ value of 1.49 µM DHCholEP (inset, Figure 4B).

#### 2.3.2. LtP and J774A.1 Macrophages

**Figure 5:**
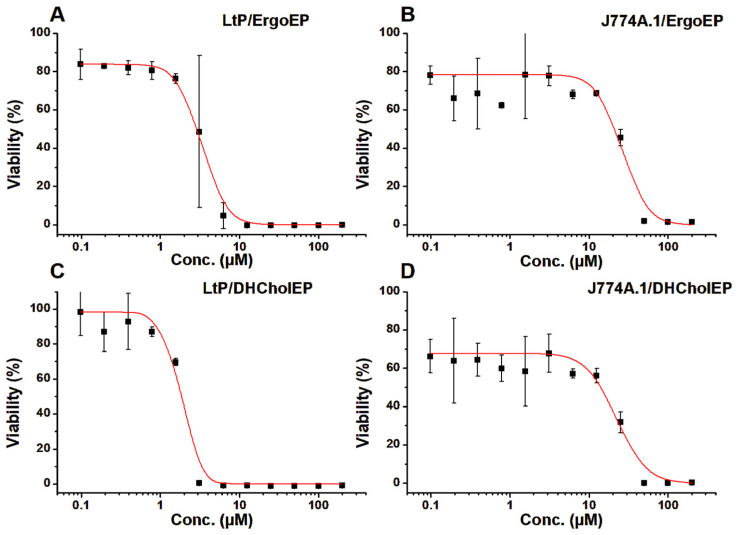
Effects of ErgoEP and DHCholEP on the viability of *L. tarentolae* promastigotes (LtP) and J774A.1 macrophages. Representative examples of viability–concentration curves are shown for LtP (**A**,**C**) and J774A.1 (**B**,**D**). Each data point represents the mean ± SD of duplicates from a single culture batch, resulting in IC_50_ values of 3.3 ± 0.1 μM (**A**), 26.2 ± 2.8 µM (**B**), 1.8 ± 0.1 µM (**C**) and 22.2 ± 2.2 µM (**D**), respectively.

Given that, for mechanistic experiments, *Leishmania tarentolae* promastigotes (LtP) and J774A.1 macrophages are more appropriate than LdP and MPM, we studied the influence of sterol EPs, parent sterols and related compounds on these cell types (Figure 5, Table 4).

Both sterol EPs were more toxic to LtP than towards J774A.1 macrophages. In contrast to MPM, sterol EPs also demonstrated some cytotoxicity towards J774A.1 macrophages (Figure 5). The average IC_50_ values from several cell batches are listed in Table 4.

The antileishmanial activity of sterol EPs was slightly lower than that of Pen, but with a higher selectivity for LtP over J774A.1 macrophages (Table 4). Sterol EPs were always more toxic to LtP than the corresponding parent sterols. In addition, compounds which interfere with *Leishmania* lipid metabolism were studied. Miltefosine (Mil), interfering in *Leishmania* phospholipid metabolism, showed strong activity against LtP and low toxicity for J774A.1 macrophages. Similarly, AmpB, interacting with ergosterol-related molecules in LtP membranes, showed IC_50_ values in LtP far below 1 µM and moderate toxicity in J774A.1 macrophages. Miconazole (MiAz), which is known to act on fungal sterol synthesis, also showed activity against LtP. Other compounds, such as pentamidine (Pen), antimycin A (AA) and Art showed IC_50_ values in the range expected from previous experiments. In both *Leishmania* species, sterol EPs (ErgoEP, DHCholEP) demonstrated significantly higher toxicity compared to their corresponding non-EPs (Ergo, DHChol). This observation contrasts with the activation patterns of both EPs and non-EPs into radicals, as depicted in Table 1 and Table 2, and Figure 2. To investigate the mechanism underlying the activity of sterol EPs (ErgoEP, DHCholEP), comparing them with non-EPs (Ergo, DHChol) as negative controls proved uninformative. This is because non-EPs can also generate radicals, albeit less efficiently. Consequently, subsequent mechanistic experiments focused exclusively on sterol EPs (ErgoEP, DHCholEP).

### 2.4. Interference of Antioxidants and Iron Chelators with Antileishmanial Effects

Since sterol EPs were demonstrated to react with Fe^2+^ and to produce carbon- and oxygen-centered radicals, the IC_50_ values of sterol EPs were evaluated in the presence of radical scavengers like thiol antioxidants, e.g., N-acetylcysteine (NAC). For comparison, they were also studied for AmpB. NAC did not display any toxicity up to 2 mM, and this concentration was used in this assay.

**Figure 6:**
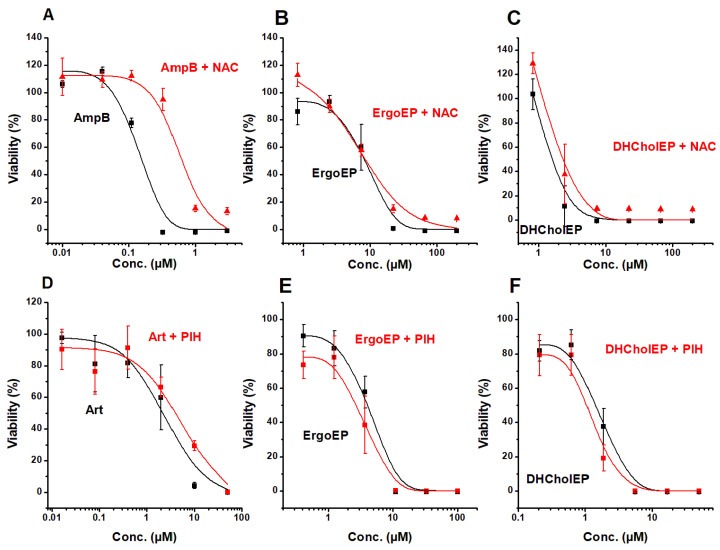
Influence of N-acetylcysteine (NAC, 2 mM) and pyridoxal isonicotinoyl hydrazone (PIH, 50 µM) on the antileishmanial effect of ergosterol endoperoxide (ErgoEP, (**B**,**E**)), dehydrocholesterol endoperoxide (DHCholEP, (**C**,**F**)) and reference compounds for NAC amphotericin B (AmpB, (**A**)) and for PIH artemisinin (Art, (**D**)). Data represent mean ± SD from triplicates. The black curves represent LtP without NAC/PIH, and the red lines indicate the inclusion of NAC or PIH. (**A**–**C**) IC_50_ values were: AmpB 0.114 µM without NAC and 0.569 µM with NAC; ErgoEP 8.33 µM without NAC and 7.27 µM with NAC; DHCholEP 2.25 µM without NAC and 2.4 µM with NAC. (**D**–**F**) IC_50_ values were: Art 2.29 µM without PIH and 4.85 µM with PIH; ErgoEP 4.68 µM without PIH and 4.31 µM with PIH; DHCholEP 1.83 µM without PIH and 1.66 µM with PIH.

The strong influence of NAC on the antileishmanial effect of AmpB was evident (Figure 6A) as the viability–concentration curve shifted, resulting in a five-fold-higher IC_50_ in the presence of NAC (Table 5). In contrast, the viability–concentration curves of ErgoEP and DHCholEP did not show any impact of NAC on their antileishmanial activity (Figure 6B,C, Table 5). This observation suggested that sterol EPs generated radicals in LtP at places at which NAC is not able to interfere. Due to the very hydrophilic properties of NAC and the very lipophilic properties of sterol EPs, this seems to be a likely reason. An alternative explanation would be that sterol EPs do not produce radicals in LtP as they fail to be activated by Fe^2+^. The effects of the iron chelator PIH on the antileishmanial activity are shown in Figure 6 and Table 5. For Art as EP, the IC_50_ value in LtP is shifted to higher values (180.5% of control) in the presence of PIH, suggesting that chelatable iron is involved in the antileishmanial mechanism of Art. In contrast, for both ErgoEP and DHCholEP, no increase, rather a slight decline, of the IC_50_ values was observed in the presence of PIH. Therefore, in spite of the fact that Art, ErgoEP and DHCholEP can react with Fe^2+^ in chemical systems, this appears not to play a major role in the antileishmanial mechanism of sterol EPs. The predictions of the lipophilicity AlogP values (BioViaDraw 2021 V21.1, Dassault Systemes, Vélizy-Villacoublay, France) for Art, ErgoEP and DHCholEP are 1.39, 5.58 and 5.78, respectively. This suggests a much stronger lipophilicity of sterol EPs than that of Art. This would favor interaction with heme-containing enzymes, such as cytochrome P450 isoenzymes or mitochondrial cytochromes (which are not influenced by PIH).

### 2.5. Interference with Mitochondrial Functions

As there are hints that sterols in *Leishmania* are not only important for the plasma membrane but also intracellular organelles [27], such as the endoplasmic reticulum and mitochondria, we studied the influence of sterol EPs on mitochondrial functions in LtP.

**Figure 7:**
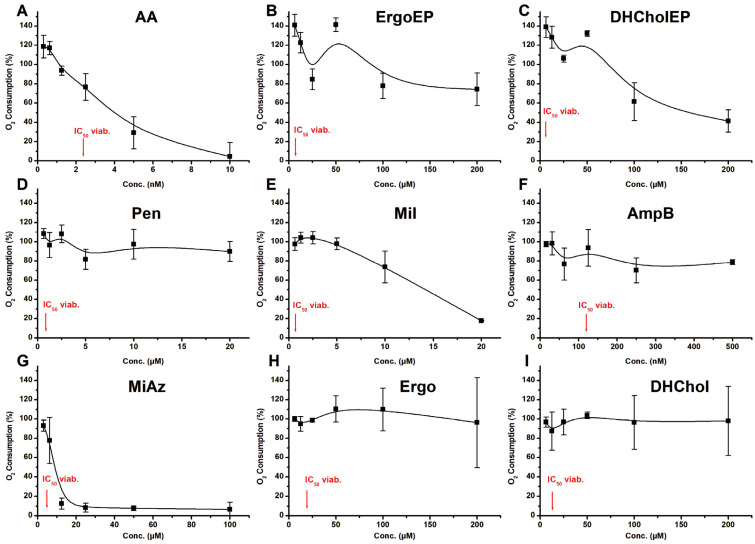
Influence of sterol EPs and other compounds on the oxygen consumption of LtP after 1 h preincubation. Compounds tested were (**A**) antimycin A (AA), (**B**) ergosterol endoperoxide (ErgoEP), (**C**) dehydrocholesterol endoperoxide (DHCholEP), (**D**) pentamidine (Pen), (**E**) miltefosine (Mil), (**F**) amphotericin B (AmpB), (**G**) miconazole (MiAz), (**H**) ergosterol (Ergo) and (**I**) dehydrocholesterol (DHChol) at concentrations around and above their IC_50_ values for viability. Data represent mean ± SD of four individual replicates for each concentration.

In a first step, the inhibition of oxygen consumption was studied after 1 h in the presence of different compounds (Figure 7). As expected, AA inhibited LtP respiration already in the nM range. In contrast, ErgoEP and DHCholEP failed to reach complete respiration inhibition even at concentrations as high as 200 µM. Their non-EP analogs Ergo and DHChol were even less effective. Nevertheless, slight inhibition was visible at high concentrations, suggesting a slow time-dependent inhibition mechanism for sterol EPs. Among typical antileishmanial agents, only Mil, not Pen or AmpB, caused respiratory inhibition of LtP. MiAz showed respiratory inhibition at low µM concentrations. This suggests that the major mode of action of antileishmanial compounds can be dependent or independent of their mitochondrial effects.

**Figure 8:**
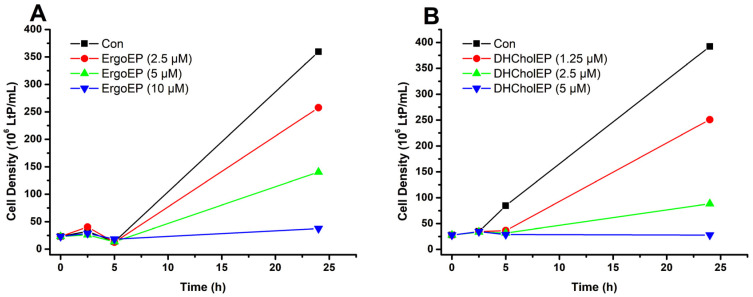
Representative growth curves of LtP (22 × 10^6^/mL) during a 24 h incubation with (**A**) ErgoEP (2.5 µM, 5 µM, 10 µM) and (**B**) DHCholEP (1.25 µM, 2.5 µM, 5 µM). Control LtP (Con) were incubated with the volume of ethanol corresponding to the vehicle of the highest sterol EP concentration. The cell density of LtP of each batch was measured after 2.5, 5 and 24 h.

To perform oximetry experiments after 24 h incubation, a sufficient number of LtP was required. Therefore, we performed LtP batch incubations with control LtP (incubated with vehicle) and LtP incubated with three different sterol EP concentrations and followed their growth over 24 h (Figure 8). From the cell batches with different sterol EP concentrations, we selected samples with clearly visible growth inhibition but a sufficient number of cells for subsequent oximetry experiments (for ErgoEP incubations with 2.5 µM and for DHCholEP 1.25 µM).

**Figure 9:**
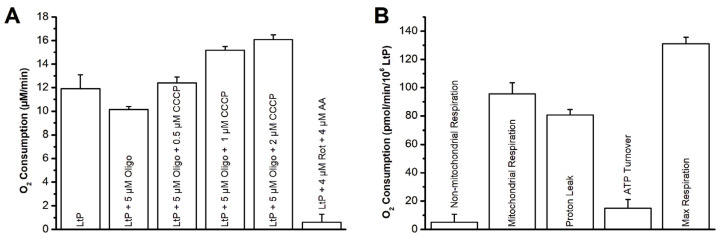
An example of oxygen consumption rates (OCR) of LtP in the absence and presence of mitochondrial inhibitors and uncoupler. The OCR was obtained from oxygen concentration–time curves of LtP incubations in Oxoplates. (**A**) Absolute OCR values (µM/min) and (**B**) bioenergetic parameters normalized to the cell number (pmol O_2_/min/10^6^ LtP). Data are mean ± SD of six wells (*n* = 6) from one cell batch.

To elucidate time-dependent effects of sterol EPs on LtP, we performed oximetry after short incubation (40 min) with high sterol EP concentration (362 µM) and batch incubations of LtP over 24 h with sterol EP concentrations around their respective IC_50_ values (1.25–10 µM). The resulting oxygen concentration–time curves were analyzed, and linear regression was performed on all data points above the anaerobic threshold to determine the oxygen consumption rates (OCR) for each well. Specifically, Figure 9A displays the absolute oxygen consumption values (µM/min) for LtP. With a set of mitochondrial inhibitors (oligomycin (Oligo), antimycin A (AA), rotenone (Rot)) and the uncoupler carbonyl cyanide 3-chlorophenylhydrazone (CCCP), we obtained a comprehensive set of bioenergetic parameters for LtP respiration (Figure 9B): (basal) mitochondrial respiration, proton leak, ATP turnover, maximal respiration and non-mitochondrial respiration normalized to cell number (pmol O_2_/min per million LtP).

The sterol-EP-treated LtP (40 min or 24 h) were compared to corresponding control LtP in oximetry experiments for ErgoEP (Table 6) and DHCholEP (Table 7). Derived bioenergetic parameters were calculated according to Brand and Nicholls [28].

The data indicate a statistically significant decrease (*p* < 0.05) of mitochondrial respiration and maximal mitochondrial respiration of LtP in the presence of ErgoEP (362 µM, 40 min). These data demonstrate a significantly increased (*p* < 0.05) coupling efficiency for LtP in the presence of ErgoEP (2.5 µM over 24 h), decreased mitochondrial respiration, decreased proton leak, a decreased portion of mitochondrial respiration related to the proton leak and an increased portion of the mitochondrial respiration used for ATP synthesis.

In analogy to oximetry experiments with ErgoEP, short-term incubations were performed for LtP with and without DHCholEP. Qualitatively, respiratory parameters behave similarly with respect to added mitochondrial inhibitors. Mitochondrial respiration in the presence of DHCholEP is slightly lower than in control LtP. For LtP incubated over 24 h with 1.25 µM DHCholEP, a significant increase (*p* < 0.05) of the coupling efficiency, increase of the respiratory control ratio, reduced mitochondrial respiration, reduced proton leak, increased ATP turnover, a decreased portion of the mitochondrial respiration associated with the proton leak and an increased portion of the mitochondrial respiration due to ATP turnover were observed.

### 2.6. Mitochondrial Superoxide Production

**Figure 10:**
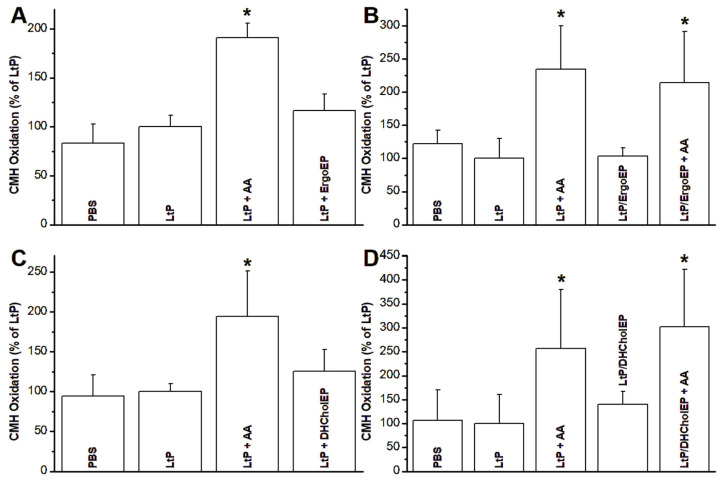
Relative changes of CMH oxidation of LtP (250 × 10^6^/mL) incubated with ergosterol endoperoxide (ErgoEP, 362 µM) for 20 min (**A**), ErgoEP (2.5 µM) for 24 h (**B**), dehydrocholesterol endoperoxide (DHCholEP, 362 µM) for 20 min (**C**) and DHCholEP (1.25 µM) for 24 h (**D**). Reference values with LtP sample type represent 100% for each incubation. Antimycin A (AA, 0.36 µM) was added directly before the EPR measurement. The bars represent mean ± SD of four cell batches (*n* = 4). For each sample type, four replicates were measured per batch. * Indicates statistically significant differences vs. LtP (*p* < 0.05).

The production of mitochondrial superoxide radicals is governed by several factors, notably the functionality of electron transfer complexes and the integrity of mitochondrial membranes. Superoxide radical formation in LtP after both short-term and long-term incubations with sterol EPs was investigated. For short-term incubations, a concentration of 362 µM sterol EP for 20 min was employed—a level considerably higher than the IC_50_ values derived from viability assays. In contrast, for long-term exposures (24 h), sterol EP concentrations were adjusted to approximate their respective IC_50_ values (1.25–10 µM). Superoxide radicals were detected via the one-electron oxidation of the cyclic hydroxylamine CMH, leading to the formation of a stable nitroxide radical (CM^●^) that was quantified using EPR spectroscopy. Measurements were performed in cell batches that were resuspended in PBS and normalized to equivalent cell concentrations (250 × 10^6^ LtP/mL). CMH oxidation was measured in both groups in the presence and absence of AA, with an additional control experiment performed using PBS without cells. To facilitate comparisons across different cell batches, the CMH oxidation rate in LtP was normalized to 100%, and all other results were expressed as a percentage relative to this control (Figure 10).

In short-term incubations of LtP with sterol EPs (Figure 10A,C), AA induced a statistically significant increase (*p* < 0.05) in CMH oxidation and, hence, superoxide radical formation. In contrast, both ErgoEP and DHCholEP showed only slightly elevated superoxide production. However, the increase was not statistically significant (*p* > 0.05) compared to the LtP controls. Long-term incubation data indicate that, despite of growth inhibition by ErgoEP and DHCholEP at concentrations close to the IC_50_ value, the superoxide production of these EP-incubated cells was only slightly elevated and similar to control LtP (Figure 10B,D). Both LtP and LtP incubated with sterol EPs after 24 h could still be stimulated by AA to a CMH oxidation, which was significantly different from that of control LtP.

### 2.7. Interference in Sterol Synthesis

**Figure 11:**
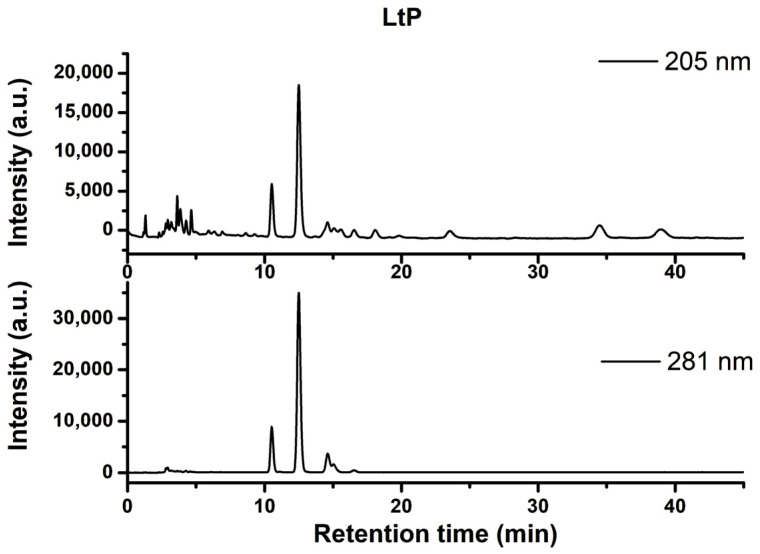
HPLC traces obtained from an extract of LtP 24 h after passage of the cell culture. The trace at 205 nm shows numerous peaks, with a main peak at 12.5 min retention time. A corresponding trace at 281 nm (which is more selective for sterols with conjugated double bonds) shows only three major peaks, with the largest peak at 12.4 min.

The involvement of various CYP enzymes as possible targets of sterol EPs in the sterol biosynthesis pathway of *Leishmania* prompted us to study the influence of sterol EPs on the ergosterol synthesis in LtP. To explore the sterol synthesis in LtP, a combined saponification/extraction method with subsequent HPLC analysis was developed. For the identification of these HPLC peaks, a set of sterol standards was analyzed (Table 8). In a first step, LtP without inhibitors were analyzed (Figure 11). The method required at least 30 mg LtP wet weight (corresponding to about 1600 × 10^6^ LtP). Therefore, incubation of LtP with and without compounds was done in 15 mL batches in culture medium starting with about 40 × 10^6^ LtP/mL, and analysis was performed after 24 h.

From the set of standards, significant amounts only of 5-dehydroepisterol (5-DHE) and Ergo and traces of Chol were detected in native LtP. By addition of standards to LtP extracts, the major peak at 12.4 min in the 281 nm and 205 nm channels was assigned to 5-DHE, and a minor peak at 14.5 min was assigned to Ergo. Hence, 5-DHE and not Ergo is the major product of the sterol synthesis in LtP. For quantification of sterol synthesis in LtP in the presence of compounds, the peak area at 281 nm was compared to a known concentration of a 5-DHE standard and finally expressed as a 5-DHE percentage of LtP pellet wet weight.

**Figure 12:**
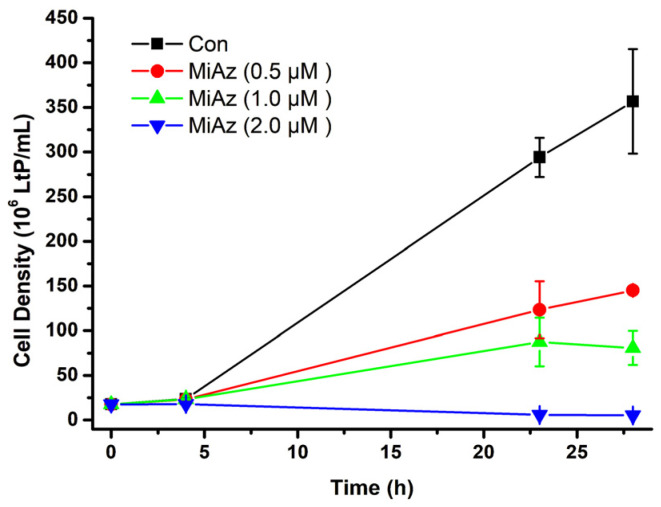
Growth of LtP in the absence (Con) and presence of different concentrations of miconazole (MiAz). Cell density was obtained from photometric measurements of the cell suspension at 600 nm. Data represent mean ± SD of three parallel batch incubation tubes.

Since at least 20–30 mg wet weight of LtP pellets was required for analysis of the resulting sterol content, triplicate parallel 15 mL batch incubations for 24 h in the absence and the presence of compounds were started per concentration. The concentrations of compounds were chosen around the IC_50_ values obtained from viability assays. Initially, the influence of inhibitors on LtP growth was followed by measuring the OD at 600 nm up to 24–28 h (as shown for MiAz, Figure 12).

At the end of each batch incubation, the LtP suspensions were centrifuged, and the resulting pellets were weighed, extracted and analyzed by HPLC for their 5-DHE content.

**Figure 13:**
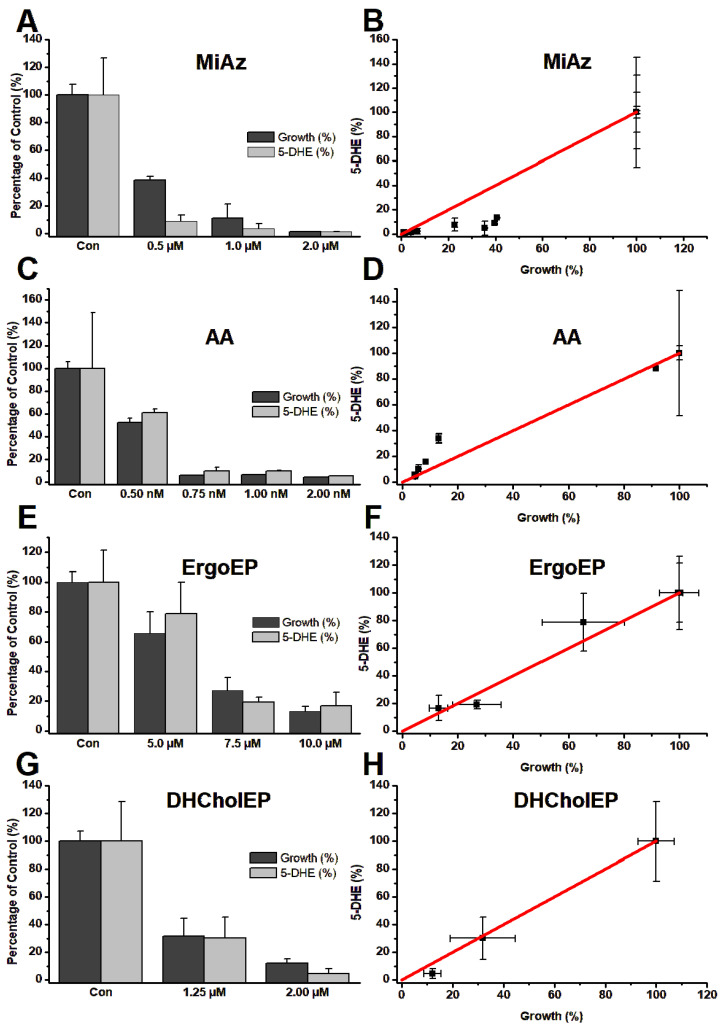
Influence of miconazole (MiAz; (**A**,**B**)), antimycin A (AA; (**C**,**D**)), ergosterol endoperoxide (ErgoEP; (**E**,**F**)) and dehydrocholesterol endoperoxide (DHCholEP; (**G**,**H**)) on LtP growth and 5-DHE content of LtP. Batch incubations of LtP were performed without or with added compounds in concentrations around their respective IC_50_ values. The final cell number after 24–28 h was used to determine growth as percentage of control samples. In the resulting LtP pellets, the 5-DHE content as percent wet weight was analyzed. The 5-DHE content in relation to the control LtP was expressed in % in the graphs. Left-hand graphs represent the mean ± SD of all batch incubations, while the right panel shows the correlation between final growth in % and the relative 5-DHE content in % for individual samples of batch incubations. Each data point represents mean ± SD from one to three tubes within a batch incubation.

For each tested compound, four batch incubations per concentration were performed. The resulting data were averaged and displayed as percentage of the control samples and illustrate the correlation between LtP growth and 5-DHE content (Figure 13).

In general, all compounds, if applied in concentrations around their IC_50_, caused a significant retardation of LtP growth. In parallel, the 5-DHE content (determined in % wet weight of LtP) decreased with increasing compound concentration (Figure 13A,C,E,G). However, the 5-DHE content is not directly proportional to growth retardation. Specifically, there are more subtle differences between the compounds (Figure 13B,D,F,H). For MiAz, it is clearly visible that only very low 5-DHE content was detectable if growth was significantly inhibited, suggesting that 5-DHE biosynthesis declines faster than growth. In contrast, for AA, which also reduces the 5-DHE content, still over-proportionally high amounts of remaining 5-DHE are detected at strong growth inhibition, suggesting that AA diminishes 5-DHE production less effectively than MiAz (Figure 13B,D). For ErgoEP and DHCholEP, decrease of growth and decrease of 5-DHE content proceed rather in parallel (Figure 13F,H), suggesting an interaction with 5-DHE synthesis stronger than for AA but not as effective as MiAz.

## 3. Discussion

Leishmaniasis remains a complex global health issue due to its diverse clinical manifestations, various causative parasites, multiple zoonotic reservoirs and regionally differing treatment options with varying success rates [4,29]. Effective, affordable and convenient treatment options are not consistently available [30]. Additionally, global warming is expanding the endemic regions of insect vectors, including areas in Europe [30]. The spread of human settlements into regions endemic to the sandfly vector further exacerbates the problem [31]. These challenges underscore the urgent need for new treatment strategies [32], which are being pursued through approaches such as mass screening of natural and synthetic compounds and the targeted development of promising compound families [33].

For *Leishmania*, particularly in their intracellular amastigote stage, import of iron and heme from host macrophages or the extracellular milieu is essential for survival [18]. Unlike mammalian cells, *Leishmania* lack cytosolic ferritin-like iron storage proteins [34], making them reliant on continuous turnover of iron and heme. This exposes free iron and heme to oxidants, which catalyze reactive oxygen species formation and trigger parasite cell death [35]. The absence of catalase in the *Leishmania* genome further exacerbates this vulnerability [36]. As a result, a portion of imported iron and heme that is not immediately directed toward the biosynthesis of iron-containing proteins accumulates in the cytosol, partly as part of the labile iron pool [15].

To target intracellular amastigotes effectively, candidate drugs must at least partially resist the macrophage’s antioxidant defenses—glutathione peroxidases, glutathione-S-transferases and catalase. EPs, which are not produced by mammalian cells, offer a promising strategy by generating potent oxidants. Consequently, several research groups have investigated EPs as antileishmanial agents [15,16,17,37,38]. The current work focuses on a subclass of EPs that has not yet been explored in depth for antileishmanial activity: sterol-derived EPs.

Sterols, found throughout eukaryotes, are polycyclic organic molecules bearing diverse functional groups. In mammals, cholesterol is the predominant sterol, whereas ergosterol fulfills this role in fungi and simpler eukaryotes. Owing to their pronounced lipophilicity, sterols preferentially localize within lipid bilayers, and their biosynthetic pathways intersect with those of numerous signaling lipids.

The susceptibility of a sterol to oxidation hinges on its molecular architecture. Those containing conjugated double bonds in the B-ring, most notably ergosterol and 7-dehydrocholesterol, readily undergo singlet-oxygen addition, rendering them particularly prone to oxidative modification.

In mammalian tissues, DHChol serves as the immediate precursor to cholesterol. In skin, ultraviolet irradiation converts DHChol into previtamin D_3_, while 7-dehydrocholesterol reductase can enzymatically reduce it to cholesterol [39]. Under normal physiology, DHChol levels remain low; however, impaired cholesterol biosynthesis in Smith–Lemli–Opitz syndrome leads to DHChol accumulation [40].

Ergosterol is the principal lipophilic membrane sterol in fungi and protozoa. Functionally analogous to cholesterol, it regulates membrane fluidity, modulates membrane-bound enzymes and ion channels and supports related cellular processes [41]. Consequently, ergosterol or ergosterol-like sterols constitute the major sterol fraction in these biological membranes.

Upon reaction with singlet oxygen, these sterols are converted into their corresponding EPs, which are stable and can be isolated in pure form. ErgoEP first drew biomedical attention when it was identified as a normal membrane component in several mushroom species [10]. Subsequent studies showed that ErgoEP exerted broad cytotoxicity with a marked preference for tumor cells [42,43]. Importantly, it demonstrated minimal toxicity toward non-transformed cells, including the J774A.1 mouse macrophage cell line and human lung fibroblasts. In malignant cells, ErgoEP suppresses STAT-family proteins—key drivers of proliferation—lowers vascular endothelial growth factor levels and exhibits antiangiogenic activity [44].

Another suggested target of ErgoEP is the Foxo3 transcription factor: ErgoEP activates Foxo3, leading to AKT and c-Myc inhibition, tumor growth arrest and apoptosis induction [45]. Beyond its anticancer profile, ErgoEP also shows antifibrotic, antiarteriosclerotic, immunosuppressive and anti-inflammatory effects in mammalian tissues.

The antimicrobial spectrum of ErgoEP spans bacteria, fungi, viruses and protozoa [10]. Of particular note is its activity against *Mycobacterium tuberculosis* [46]. It also inhibits protozoal parasites such as *Trypanosoma cruzi* and *Plasmodium* species [10]. Although the exact mechanisms remain to be fully elucidated, parallels with its action in cancer cells suggest induction of oxidative-stress signaling in protozoa. Antitrypanosomal effects are attributed to direct membrane disruption [47] and free-radical reactions initiated by ErgoEP [48]. In silico docking further predicts strong binding to *Trypanosoma* sterol-biosynthesis enzymes—sterol 14α-demethylase (CYP51), squalene synthase and lanosterol 14α-demethylase [48]. To date, only limited studies have addressed ErgoEP’s direct effects on *Leishmania* species [25].

Oxidation products of Chol and DHChol have garnered interest in studies of oxidative stress and related pathologies in mammalian tissues. In those investigations, however, DHCholEP was either not detected or its isolated bioactivity was not assessed [39]. To date, there are no reports of endogenous DHCholEP in animal tissues. The chemical synthesis of DHCholEP was described in the course of efforts to enhance the anticancer properties of ErgoEP analogs [49]. Its specific antimicrobial activity has not yet been investigated.

The primary aim of this study was thus to evaluate the antileishmanial potential and dissect the mechanism of action of ErgoEP and DHCholEP. Although ErgoEP has been purified from natural sources [24,44,50], we opted for chemical synthesis to secure sufficient amounts of these EPs. Both EPs were generated by a photochemical [4 + 2]-cycloaddition of ^1^O_2_ to the conjugated diene moiety in their parent sterols Ergo and DHChol using MB as photosensitizer (Figure 1). Following chromatographic purification and verification by NMR spectroscopy, the isolated EP fractions exceeded 99% peroxide content, though containing traces of dehydroendoperoxy derivatives as byproducts.

As an initial biochemical characterization, we probed the reactivity of sterol EPs and their parent sterols towards Fe^2+^ using the XO assay. In a MeOH/H_2_O mixture, both sterol EPs and their parent sterols rapidly oxidized Fe^2+^ (Table 1). By contrast, cholesterol, despite its close structural similarity but lacking B-ring conjugation, showed no detectable reaction with Fe^2+^ (similar to the vehicle control with EtOH).

In an air-saturated ACN/H_2_O mixture, the reaction of sterol EPs (ErgoEP and DHCholEP) and their parent sterols (Ergo and DHChol) with Fe^2+^ generated multiple DMPO spin adducts detectable by EPR spin trapping (Figure 2). For parent sterols (non-EPs), this seems to be unexpected; however, taking into account that all experiments were carried out in air-saturated solutions, activation of non-EPs (Ergo, DHChol) by formed Fe^3+^ may proceed via the formation of intermediate hydroperoxides [51], which then are decomposed to observed radical species. The EPR spectral patterns of the spin adducts from ErgoEP and DHCholEP were more similar to each other than to those from Ergo and DHChol. Simulations of the experimental spectra yielded the hyperfine coupling parameters (Table 2). The detected spin adducts represent mixtures of carbon-centered radicals and oxygen-centered radicals, the latter including both peroxyl and alkoxyl species. In all tested compounds, the intensities of spin adducts from carbon-centered radicals were smaller than the combined intensities of adducts from oxygen-centered radicals, suggesting that carbon-centered radicals are secondary products of the compound degradation. The different abundance patterns of ROO^●^ vs. RO^●^ are not correlated to structural features of EPs and non-EPs, suggesting complex activation pathways. This spin adduct profile matches observations for Art and anthracene EPs [16,17] but contrasts with the case of ascaridole, where primary oxygen-centered radicals are rapidly stabilized by conversion into carbon-centered isopropyl radicals [15]. These findings indicate that diene-containing sterols and their EPs share a common iron-mediated reactivity rather than reacting according to entirely distinct mechanisms. Indeed, diene-containing sterols (Ergo, DHChol) are known from chemical literature to undergo iron activation, forming reactive intermediates with alkylating properties, a reactivity not seen for cholesterol [51,52]. Mechanistically, our data support a complex decomposition pathway initiated by one-electron reduction of the EP bridge by Fe^2+^, leading to both carbon- and oxygen-centered radicals. Such radical generation by sterol EPs could contribute to their antileishmanial mechanism. However, its biological significance may be limited by sterol phase separation between lipid membranes and the aqueous cytosol where low-molecular-iron complexes are available.

The biological effects of sterol EPs were evaluated in two complementary in vitro systems. First, we assessed antileishmanial activity against LdP and cytotoxicity in mouse peritoneal macrophages (MPM). Second, mechanistic insights were sought using LtP and J774A.1 macrophages. Both ErgoEP and DHCholEP showed pronounced antileishmanial effects in the LdP assay, with IC_50_ values in the low µM range (Figure 3A,C). In parallel cytotoxicity tests, neither sterol EP exhibited significant toxicity toward MPM up to 500 µM (Figure 3B,D). When compared to their parent sterols, each EP had a lower IC_50_ value than the corresponding sterol (Table 3). This is different from the similar chemical reactivity of sterols and sterol EPs with Fe^2+^ (Table 1). The selectivity indices ranged from 27 to 60 for sterol EPs (Table 3), reflecting minimal macrophage toxicity.

In the anti-amastigote assay using *L. donovani* inside MPM, ErgoEP and DHCholEP retained efficacy with IC_50_ values of 8.1 and 1.49 µM, respectively (Figure 4). This demonstrates that both sterol EPs can effectively cross macrophage membranes and accumulate in the phagolysosome, where they exert their antileishmanial activity.

In vitro viability assays were conducted on LtP and J774A.1 macrophages (Figure 5). The resulting IC_50_ values (Table 4) demonstrate that ErgoEP and DHCholEP inhibited LtP growth with IC_50_ values of 4.5 µM and 2.0 µM, respectively, confirming that both EPs are more active than their parent sterols and that DHCholEP is slightly more potent than ErgoEP.

When compared with other antileishmanial agents, sterol EPs exhibit only moderate potency and limited selectivity in this model. For example, the mitochondrial inhibitor AA shows an extremely low IC_50_ of 2.5 nM, yet it is unsuitable for therapy due to rapid resistance development in *Leishmania* [53]. Notably, the LtP IC_50_ for the sterol EPs in this study are comparable to those reported for the antimalarial EP artemisinin.

The IC_50_ values for ErgoEP in LdP and LdA closely match those values published by Leliebre-Lara et al. [25], although they observed higher toxicity toward MPM (IC_50_ = 43 µM). Such discrepancies likely stem from variations in culture and assay conditions, as previously discussed [54].

To investigate whether radical formation contributes to the antileishmanial action of sterol EPs, we measured LtP viability in the absence and presence of the radical scavenger NAC at non-toxic concentrations (Figure 6). If EP-derived radicals underlie parasite killing, NAC should raise the IC_50_ values, as shown for AmpB (Figure 6A, Table 5). In contrast, neither ErgoEP nor DHCholEP showed any NAC-induced shift in IC_50_ (Figure 6B,C; Table 5). This outcome implies that EP-triggered radicals are either not central to their antileishmanial effect or are formed in microenvironments inaccessible to NAC-scavenging.

Given our demonstration that low-molecular-weight Fe^2+^ complexes can reduce sterol EPs into radicals, we assessed the impact of the lipophilic iron chelator PIH on antileishmanial potency in the next step (Figure 6).

IC_50_ values of artemisinin in LtP increased markedly in the presence of PIH (Figure 6D; Table 5), confirming dependence on free iron. By contrast, PIH had no effect on the IC_50_ values of ErgoEP or DHCholEP (Figure 6E,F; Table 5). PIH is a highly effective, lipophilic chelator of low-molecular-weight iron complexes within the cytosol but does not mobilize iron from metalloproteins such as cytochromes and cytochrome P450 isoenzymes [55,56]. These data suggest that freely available Fe^2+^ may not be the primary activator of sterol EPs in cells. Instead, activation may occur in intimate association with iron-containing proteins.

To assess how sterol EPs impact mitochondrial function, we measured oxygen consumption by LtP in the presence of antileishmanial compounds including sterol EPs after 1 h incubation with increasing concentrations of sterol EPs and other antileishmanial drugs (Figure 7). As expected, the mitochondrial inhibitor AA nearly abolished respiration at low nanomolar concentrations (within the range of its IC_50_ for viability in LtP). In contrast, ErgoEP and DHCholEP required concentrations above 100 µM—far higher than their IC_50_ in viability assays—to achieve ≥50% inhibition of oxygen consumption. Their non-EP analogs (Ergo, DHChol) were even less effective. Among other antileishmanial agents tested, Pen and AmpB had no effect on LtP respiration (at their IC_50_ in viability assays), whereas Mil and MiAz each produced half-maximal respiratory inhibition at about 10 µM. While Mil’s half-maximal inhibition of oxygen consumption occurs at concentrations much higher than its IC_50_ for LtP viability, MiAz’s inhibition of oxygen consumption occurs within the same range as its IC_50_ for LtP viability. If a compound inhibits oxygen consumption at concentrations similar to its IC_50_ for LtP viability, it likely involves mitochondrial respiration inhibition as part of its antileishmanial mechanism (as observed with AA and MiAz). Conversely, if oxygen consumption inhibition occurs only at significantly higher concentrations than the IC_50_ for LtP viability, mitochondrial respiration inhibition is probably not the primary target in the compound’s antileishmanial activity (as seen with various compounds such as ErgoEP, DHCholEP, Ergo and DHChol). These data demonstrate that suppression of mitochondrial respiration in LtP after 1 h is not a prerequisite for antileishmanial activity.

To examine how sterol EPs affect LtP oxygen metabolism at their inhibitory concentrations, we set up 15 mL batch cultures of LtP with ErgoEP or DHCholEP at doses bracketing their IC_50_ values and incubated them for 24 h at 26 °C. Compound concentrations were chosen based on previous 96-well viability assays. We monitored cell density in batch incubations over time (Figure 8). After 24 h, cell counts in treated cultures ranged from 100% of the vehicle-treated control down to only a few percent at the highest sterol EP concentrations. From these data, we estimated IC_50_ ranges of 2.5–5 µM for ErgoEP and 1.25–2.5 µM for DHCholEP. These values align closely with those determined in the microplate assays, demonstrating that our batch-culture system faithfully reproduced the growth-inhibition profile seen in viability tests. Consequently, any downstream measurements on these cell batches will accurately reflect the biologically active state of the sterol EPs under antileishmanial conditions.

Typical oxygen consumption experiments conducted in a 96-well format revealed the basal respiration of LtP and the decrease in respiration in the presence of Oligo, an inhibitor of ATP synthase (Figure 9A). Upon the addition of increasing amounts of the uncoupler CCCP, a typical increase in respiration above basal respiration rates was observed. The addition of strong electron-transfer inhibitors, Rot and AA, decreased respiration to very low values, differing from macrophages which have significant non-mitochondrial respiration due to NADPH oxidases [57]. Based on these respiration rates and cell number, mitochondrial bioenergetic parameters were calculated as shown in Figure 9B.

Short-term incubations (40 min) of LtP with high concentrations of ErgoEP (Table 6) led to a significant decrease in both basal and maximal mitochondrial respiration compared to control LtP. This indicates a slight reduction in mitochondrial electron transfer by ErgoEP, which could result from the inhibitory activity of ErgoEP at mitochondrial electron transfer complexes or from a limited substrate supply to mitochondria due to partial inhibition of glycolysis. Further studies on the effects of ErgoEP at lower concentrations during long-term incubations also showed a significant decrease in mitochondrial respiration, along with a decrease in proton leak (Table 6). This suggests an increased efficiency of mitochondrial LtP functions because of ErgoEP, as evidenced by improved coupling efficiency. Additionally, the portion of oxygen consumption used for ATP production is higher, and the portion of the proton leak related to mitochondrial respiration is lower in ErgoEP-incubated LtP compared to control LtP. The mechanism by which sterol EPs and resulting membrane changes influence mitochondria can include many effects, such as modulation of the activity of electron transfer enzymes [58] and membrane carriers [59] and influence on membrane proton conductivity [60].

Short-term incubations of LtP with high concentrations of DHCholEP (Table 7) led to decreased mitochondrial respiration, maximal mitochondrial respiration and proton leak. Long-term incubations with DHCholEP at lower concentrations showed more pronounced effects (Table 7). Mitochondrial respiration and proton leak decreased, while ATP turnover increased. Additionally, the coupling efficiency and respiratory control ratio in the presence of DHCholEP were higher than in control LtP. Relative parameters indicated that a higher portion of oxygen consumption was used for ATP production and a lesser portion to compensate for the proton leak. This suggests that, similar to ErgoEP, DHCholEP leads to a higher ATP demand and a slight decline in mitochondrial respiration, possibly due to extra-mitochondrial effects, as the coupling efficiency of mitochondria in LtP is improved in the presence of DHCholEP. This surprising finding could be due to membrane changes triggered by ErgoEP and DHCholEP. It could also represent a preconditioning effect in the cell batches that survived the toxic activity of ErgoEP after 24 h, as most of these batches had a viability of more than 50%.

Another aspect of mitochondrial oxygen metabolism involves the generation of superoxide radicals. These radicals were detected through their reaction with CMH, which leads to the formation of a stable nitroxyl radical, CM^•^. This radical was subsequently identified and quantified using EPR spectroscopy.

To investigate the immediate effects of sterol EPs on LtP, short-term incubations were conducted in which LtP were exposed to sterol EPs for 20 min at concentrations approximately 100 times higher than their IC_50_ values. In contrast, long-term incubations utilized LtP derived from batch cultures that had been treated with sterol EPs at concentrations near their IC_50_ values. These conditions were chosen to assess the prolonged effects of sterol EPs at levels relevant to their antileishmanial activity.

In short-term incubations, the mitochondrial inhibitor AA elicited a significantly higher rate of superoxide release compared to untreated LtP controls (Figure 10A,C). Although high concentrations of ErgoEP and DHCholEP produced slight increases in superoxide output, these changes did not reach statistical significance versus the control LtP. This indicates that even at concentrations much higher than IC_50_ levels, sterol EPs provoke only a minimal immediate rise in superoxide production.

In long-term incubations, LtP remained fully responsive to AA, showing significantly elevated superoxide release rates (Figure 10B,D). Treatment with ErgoEP or DHCholEP under these prolonged conditions did not significantly enhance basal superoxide release, despite a modest upward trend. Furthermore, LtP pre-incubated for 24 h with low (near-IC_50_) sterol EP concentrations still generated additional superoxide upon AA stimulation to the same extent as control LtP. These findings suggest that sterol EP treatment does not compromise mitochondrial respiratory chain function.

We note, however, that our analysis may be biased by the necessity to select cell batches with ≥50% viability to ensure sufficient sample size for EPR measurements. Consequently, batches with lower viability were not assessed under the current experimental constraints.

To explore another possible mechanism of action for sterol EPs, we analyzed endogenous sterol production in *Leishmania* and its modulation by sterol EPs. Lipids are central to protozoan biology, governing energy production, membrane architecture and host–parasite interactions [20]. *Leishmania* parasites depend on ergosterol and related sterols for their sterol metabolism [19], yet the most abundant sterols they produce are 5-dehydroepisterol (5-DHE) and episterol [61]. Their distinctive lipid composition and fatty acid profiles enable immune evasion, making the sterol biosynthesis pathway a promising therapeutic target [62]. Given the structural similarity between sterol EPs and *Leishmania*’s native sterols, we hypothesized that sterol EPs might disrupt the parasites’ sterol biosynthesis.

Based on the identification of sterol compounds in LtP by chromatographic methods using respective standards (Table 8), 5-DHE was identified as major sterol in LtP, while only minor portions of Ergo were detected (Figure 11). Ergo and 5-DHE share the same molecular mass, and also their polycyclic ring moiety is identical and differentiated only by the structure of the side chain. This may explain why some publications working with GC–MS reported them as Ergo-I and Ergo-II isomers without clear structural assignment [63], while still other publications did not even report the presence of both isomers [19]. For *L. infantum*, high amounts of both isomers partially varying with the virulence of the strain were reported [63]. Torres-Santos et al. reported approximately equal amounts of Ergo and 5-DHE in *L. amazonensis* promastigotes and that their amount differently changed upon treatment with a chalcone derivative [61]. Andrade-Neto et al. reported 5-DHE with 83.4% of total sterols as the major sterol in *L. amazonensis* promastigotes and that its portion dropped to 4.15% upon treatment with 4 µM MiAz [64]. Sterol profiling in *L. donovani* and *L. tarentolae* promastigotes showed that 5-DHE content was strongly downregulated upon the presence of azole-type inhibitors, while 14-methylzymosterol and 14-methylfecosterol were increasingly accumulated under these conditions, indicating a clear relationship between 14α-demethylase (CYP51) inhibition and the sterol profile.

To assess how sterol EPs in comparison to MiAz and AA influence LtP growth and sterol composition, we performed 24 h incubations with sterol EP concentrations around their IC_50_ values alongside vehicle-treated controls (Figure 12). The growth of LtP samples incubated with each compound at the tested concentrations was significantly slowed down or completely arrested. After 24 h incubation, cell pellets were harvested for HPLC analysis. Dose-response quantification of 5-DHE showed a clear, concentration-dependent decline in 5-DHE levels across all treated samples (Figure 13). Shown in the left-hand panel of Figure 13, the inhibition of LtP growth parallels the reduction in relative 5-DHE content, indicating that higher compound concentrations limit sterol lipid production and, thus, lower their proportion in membrane lipids. As anticipated, MiAz—an azole-type inhibitor of sterol synthesis—produced a strong decrease in 5-DHE content. This is expected since azoles are known inhibitors of 14α-demethylase, an essential enzyme for leishmanial sterol synthesis [65]. By contrast, while AA, a mitochondrial inhibitor, also reduced 5-DHE levels, the effect was less obvious mechanistically. Because sterol biosynthesis is highly energy-demanding, AA-mediated mitochondrial inhibition likely retards this complex pathway earlier than other lipid metabolic processes. Furthermore, it has been reported that in fibroblasts, inhibition of the mitochondrial electron transfer has functional consequences for the mevalonate pathway and reduces the amount of cholesterol precursors [66]. A similar mechanism could be of importance for the reduction of 5-DHE in LtP upon inhibition with AA. To distinguish primary from secondary effects on sterol levels, we plotted relative 5-DHE content versus sample growth (Figure 13B,D,F,H). MiAz data points lay predominantly below the red proportionality line, consistent with direct sterol synthesis inhibition. AA points fell mostly above the line, suggesting that 5-DHE decline is secondary to growth inhibition. ErgoEP and DHCholEP data cluster near the proportionality line, indicating that their impact on 5-DHE production is more directly tied to sterol synthesis in LtP than AA but less potent than MiAz. Taken together, these results suggest that sterol EPs interfere with the sterol biosynthesis pathway—or closely related processes—critical for 5-DHE production in LtP. This hypothesis is supported by in silico inverse-docking studies in *T. cruzi*, which identified sterol 14α-demethylase (CYP51) and lanosterol 14α-demethylase as top binding targets of ErgoEP [48]. In addition, ErgoEP has been observed as a lipid signaling molecule in *Saccharomyces cerevisiae*, appearing under oxidative stress in the outer mitochondrial membrane and facilitating recognition processes for mitochondrial quality control [67]. Besides the effects sterol EPs exert on *Leishmania* itself, it is likely that they also influence the host cells and parasite–host interactions, such as inflammatory processes [68], host cell antigen presentation [69] and host cell membrane sterol composition in *Leishmania* infection [70,71,72].

## 4. Materials and Methods

### 4.1. Reagents

All chemicals were of analytical grade, unless stated otherwise. 7-Dehydrocholesterol (DHChol), cholesterol (Chol), antimycin A (AA), artemisinin (Art), brain heart infusion (BHI) powder, M199 medium, hemin, pentamidine (Pen), resazurin, xylenol orange (XO), methylene blue (MB), oligomycin (Oligo), carbonyl cyanide 3-chlorophenylhydrazone (CCCP), rotenone (Rot), 5,5-dimethyl-1-pyrroline-N-oxide (DMPO) and paraffin oil were from Sigma-Aldrich (St. Louis, MO, USA). 5-Dehydroepisterol (5-DHE) was from THP Medical Products (Vienna, Austria). Acetonitrile (ACN), acetone, KCl, K_2_HPO_4_, KH_2_PO_4_, (NH_4_)_2_Fe(SO_4_)_2_, NH_4_SCN, H_2_SO_4_, methanol (MeOH), n-hexane, petrol ether (PE), ethyl acetate, CHCl_3_, CH_2_Cl_2_, CaCl_2_, CDCl_3_, Na_2_HPO_4_, NaCl, KOH, diethylenetriaminepentaacetic acid, sodium dithionite and glucose monohydrate were from Merck (Darmstadt, Germany). Amphotericin B (AmpB) was from Cayman Chemical (Ann Arbor, MI, USA), butylated hydroxytoluene from Roche (Basel, Switzerland) and deferoxamine from Novartis (Basel, Switzerland). Penicillin–streptomycin solution was from VWR (Radnor, PA, USA), 1-hydroxy-3-methoxycarbonyl-2,2,5,5-tetramethylpyrrolidine (CMH) from Noxygen (Elzach, Germany). Dulbecco’s modified Eagle’s medium (DMEM), Roswell Park Memorial Institute (RPMI) 1640 medium, miconazole (MiAz), miltefosine (Mil), N-acetylcysteine (NAC) and fetal bovine serum (FBS) were from Thermo Fisher Scientific (Waltham, MA, USA). Ergosterol (Ergo) was from Across Organics (Geel, Belgium), ethanol (EtOH) from Scharlab (Barcelona, Spain), fetal calf serum (FCS) from Bio&SELL (Nuremberg, Germany) and yeast extract from Amresco (Solon, OH, USA). MTS (3-(4,5-dimethylthiazol-2-yl)-5-(3-carboxymethoxyphenyl)-2-(4-sulfophenyl)-2H-tetrazolium) was from Promega (Madison, WI, USA), PMS (phenazine methosulfate) from Sisco Research Laboratories (Andheri, Mumbai, India). The cDNA reverse transcription kit was from Applied Biosystems (Grand Island, NY, USA) and TRIzol reagent from Ambion (Austin, TX, USA). All reagents, instruments and analyzing software for droplet digital polymerase chain reaction (ddPCR) were from Bio-Rad Laboratories (Hercules, CA, USA). Dehydrocholesterol endoperoxide (DHCholEP) and ergosterol endoperoxide (ErgoEP) were synthesized and purified as described below.

### 4.2. Sterol EP Preparation

#### 4.2.1. Synthesis of ErgoEP and DHCholEP

The synthesis was adapted from [46,73]. For the synthesis of sterol EPs, CH_2_Cl_2_ was dried over anhydrous CaCl_2_ overnight, and a 50 mL aliquot was placed in a 100 mL two-neck round-bottom flask. Subsequently, 200 mg of respective parent sterol was added into the solvent under magnetic stirring. During the process, the solution was cooled down with ice. A constant flow of oxygen was introduced into the reaction liquid. After 1 h, 5 mg of methylene blue (MB) was added to the mixture. Afterwards, the solution was irradiated for 20 min with two tungsten filament lamps placed 25 cm above the flask under a constant supply of oxygen. During this process, 200 µL aliquots were taken at different periods of time for evaluation by thin-layer chromatography (TLC). The liquid reaction mixtures were combined and stored at 4 °C until further purification of the products.

#### 4.2.2. Thin-Layer Chromatography (TLC)

To evaluate the conversion process of sterols to their corresponding EPs, TLC was used. Using glass capillary tubes, 5 µL samples of the aliquots were applied onto a 10 × 10 cm TLC plate (TLC aluminum sheets with silica gel 60 F254, Art. No. 1.05554.0001, Merck, Darmstadt, Germany) and allowed to dry for at least 15 min. For equilibration, 30 mL of mobile phase (CHCl_3_ (97% *v*/*v*)/MeOH (3% *v*/*v*)) was placed in a TLC chamber (VWR, Radnor, PA, USA). Then, the plate was developed and the plate was left for 20 min to dry. Afterwards it was illuminated with a high-pressure mercury UV-lamp with a 340 nm short-pass filter to visualize the spots. Spots of sterol EPs were visualized by Fe^2+^/SCN staining of the plates using a NH_4_SCN/(NH_4_)_2_Fe(SO_4_)_2_ mixture in acetone/H_2_O (14:1 *v*/*v*). Typical R_f_ values were: Ergo, 0.4; DHChol, 0.36; DHCholEP, 0.22; ErgoEP, 0.23.

#### 4.2.3. Vacuum Liquid Chromatography (VLC)

The three individual reaction batches were combined (three in total) and further purified using VLC. Initially, the solvent of the pooled fractions was subsequently evaporated using Rotavapor™ (Büchi, Flawil, Switzerland) until approximately 50 mL of the solvent was left in the flask. About 2 g of Celite^®^ 545 (VWR, Radnor, PA, USA) was added to the mixture, and the evaporation process was continued to dryness. For VLC, a glass column (20 × 400 mm, 125 mL capacity) with a sintered frit at the bottom and a vacuum connector (Lenz, Wertheim, Germany) was used. The column was loaded starting with a filter paper disc and then silica gel (0.02–0.045 mm) (Carl Roth, Karlsruhe, Germany) up to a height of about 18 cm. Then, the silica gel was compressed and covered with another filter paper disc and the Celite containing the crude products. A dripping funnel was placed on top of the filled column, and a vacuum pump was connected to the column. Then, the stationary phase was saturated with 60 mL of PE. Gradient elution of the column was performed using different volumes of PE and ethyl acetate. The fractions were collected in 20 mL glass tubes. From each fraction, 1 mL was transferred to 1.5 mL Eppendorf tubes and placed on a thermomixer (Eppendorf, Hamburg, Germany) at 37 °C with open lids until the solvents were completely evaporated. The residue was resuspended with 50 µL of ethyl acetate and subsequently analyzed using TLC, UV-illumination and Fe^2+^/SCN staining. The glass tubes, which contained fractions with the highest amount of desired product, underwent evaporation using nitrogen and were then analyzed by nuclear magnetic resonance (NMR).

#### 4.2.4. NMR

^1^H NMR spectra were recorded on a Bruker Avance II 400 (Rheinstetten, Germany) equipped with a 5 mm N_2_-cooled cryoprobe head (Prodigy) with z-gradients at room temperature. Resonance frequencies were 400.13 MHz for ^1^H and 100.63 MHz for ^13^C. Samples (approx. 20 mg) were dissolved in 0.6 mL of CDCl_3_ (99.8% D). Chemical shifts are given in δ ppm values, with reference to residual solvent signal (CDCl_3_, 7.26 ppm for ^1^H, 77.0 ppm for ^13^C). Structure elucidation and resonance assignment of the ergosterol derivatives was performed by acquisition and analysis of different one- and two-dimensional spectra (^1^H, ^13^C, COSY, HSQC, HMBC) using TopSpin 3.2.7 or higher (Bruker) software. For evaluation of ErgoEP sample purity, the following signal integrals were used at (δ): 6.59, 6.50 and 5.57 for DHErgoEP (dehydroergosterol endoperoxide), ErgoEP and Ergo, respectively. For DHCholEP samples, the following signal integrals were used at (δ): 6.60, 6.50 and 5.57 for TDHCholEP (tetradehydrocholesterol endoperoxide), DHCholEP and DHChol, respectively. Integrals were summed and set to 100%, and respective portions of compounds were expressed in percentages. Accordingly, the ErgoEP preparation contained 90.2% ErgoEP, 9.4% DHErgoEP and 0.3% Ergo (>99% EP content). The DHCholEP preparation contained 95.9% DHCholEP, 3.0% TDHCholEP and 1.0% DHChol (99% EP content). NMR data of the compounds are summarized in the Supplementary Information in Tables S1–S3 and Figures S3–S14.

### 4.3. XO Assay

To conduct the xylenol orange (XO) assays, an organic peroxide reagent (0.125 mM XO, 4 mM butylated hydroxytoluene, 90% MeOH) was prepared. Immediately before the experiments began, the organic peroxide reagent was mixed with a freshly prepared iron stock (25 mM (NH_4_)_2_Fe(SO_4_)_2_, 2.5 M H_2_SO_4_) in the ratio of 1:100, resulting in a final concentration of 250 µM Fe^2+^. Organic peroxide reagent (1 mL) mixed with iron stock was placed in a 1.5 mL disposable cuvette (BRAND, Wertheim, Germany). Then, 5 µL of the respective compound stock (20 mM) was added (100 µM final concentration). The optical density was measured for 20 min in 20 s intervals in the range of 400 nm–700 nm using a MS1501 UV–VIS diode array spectrophotometer (Shimadzu, Kyoto, Japan). MeOH/H_2_O (9:1 *v*/*v*) was used as reference. For the kinetic evaluation, the increase of the absorption at a wavelength of 560 nm was used. The initial slope of the XO/Fe^3+^ complex formation, due to the reaction of the compounds with Fe^2+^, was converted to the concentration change (nmol/min/mL) via the extinction coefficient of XO/Fe^3+^ (15,000 L·mol^−1^·cm^−1^).

### 4.4. Spin Trapping in a Chemical System

For electron paramagnetic resonance (EPR) spin trapping, 100 µL of ACN/H_2_O (1:1 *v*/*v*) with 20 mM DMPO, 900 µM sterols/sterol EPs and 900 µM (NH_4_)_2_Fe(SO_4_)_2_ were prepared. After an incubation period of 2 min at 25 °C, 50 µL of the mixture was aspirated into a disposable glass micropipette (BLAUBRAND intraMARK, BRAND, Wertheim, Germany) and inserted into the split ring resonator (Bruker MD5) of the EPR spectrometer (Bruker Digital Upgrade EMX, Rheinstetten, Germany). For the measurements, the following parameters were used: microwave frequency 9.685329 GHz; microwave power 20 mW; modulation frequency 100 kHz; modulation amplitude 1 G; time constant 0.01 s; center field 3449 G; sweep width 100 G; scan time 60 s; attenuation 70 dB; scans 10; measurement temperature 25 °C.

The obtained spectra of the EPR spin trapping experiments were simulated with the software WINSIM 2002 from the P.E.S.T. (Public Electron Paramagnetic Resonance Software Tools) library of the National Institute of Environmental Health Sciences (NIEHS-NIH, Research Triangle Park, NC, USA) [74] until a sufficient fit was achieved, and the derived coupling parameters were used for assignment of the spin adducts (complete simulation data sets are given in the Supplementary Information (Table S4, Figures S15–S18)).

### 4.5. Maintenance of Mice

Swiss albino mice (20–25 g) of either sex from the State Centre for Laboratory Animal Breeding (under West Bengal Livestock Development Corporation Ltd., Kolkata, India), Kalyani (Registration No. 1913/GO/Bt/S/16/CPCSEA) were housed in the animal facility of the Institute of Postgraduate Medical Education and Research (IPGME&R), Kolkata, India under controlled temperature (22 ± 5 °C) and humidity (30–70%) and a 12 h light/dark cycle. The animals were provided with standard rodent chow and water ad libitum. All the experiments were approved by the Institutional Animal Ethics Committee of IPGME&R, Kolkata (Registration No. 5544/GO/Re/S/02/CPCSEA). To obtain murine peritoneal macrophages, animals underwent cervical dislocation and were terminated as per CPCSEA (Committee for the Purpose of Control and Supervision of Experiments on Animals) recommendations.

### 4.6. Cell Culture

#### 4.6.1. *Leishmania tarentolae* Promastigotes

Mechanistic experiments were carried out with *Leishmania tarentolae* promastigotes (LtP) as a model system (strain P10, Jena Bioscience, Jena, Germany). For the cultivation of the cells, BHI medium (37 g BHI/L, pH 7.4) was supplemented with 5 mg/L hemin, 25,000 IU/L penicillin G and 25 mg/L streptomycin. The LtP were cultivated in 50 mL TubeSpin^®^ bioreactors (TPP Trasadingen, Trasadingen, Switzerland), and 10–15 mL culture per tube was incubated at 26 °C (Incu-Line 150R, Hassrode, VWR, Darmstadt, Germany). The cells were passaged three times a week and counted using a photometer (U-1100, Hitachi Ltd., Tokyo, Japan), measuring the optical density (OD) at 600 nm. The cell concentration was then calculated according to [75].

#### 4.6.2. *Leishmania donovani* Promastigotes

*Leishmania donovani* promastigotes (LdP) [MHOM/IN/1983/AG83] were maintained up to 10 passages at 24 °C in M199 medium (Phenol Red^+^) with 10% heat-inactivated FBS, penicillin G (50 IU/mL) and streptomycin (50 μg/mL). Cells were sub-cultured every 72 h, inoculum being 1 × 10^6^ cells/mL. For establishing ex vivo infection in murine peritoneal macrophages, 5–7-day-old stationary metacyclic promastigotes were used.

#### 4.6.3. Murine Peritoneal Macrophages

Resident murine peritoneal macrophages (not elicited by thioglycolate) were isolated from Swiss albino mice and resuspended in RPMI medium (Phenol Red^+^) supplemented with 10% FCS, penicillin G (50 IU/mL) and streptomycin (50 μg/mL). Next, peritoneal macrophages (~2 × 10^6^/mL/well in 12-well plates or 5 × 10^5^ cells/200 µL/well in 96-well plates) were allowed to adhere overnight at 37 °C and 5% CO_2_ in an incubator for subsequent experiments.

#### 4.6.4. J774A.1 Macrophages

For additional experiments, a murine macrophage cell line, J774A.1 (mouse, ATCC^®^, TIB-67™, Wesel, Germany), was used. The cultivation of the J774A.1 macrophages was performed in DMEM (high glucose, 1.5 g/L NaHCO_3_), 25,000 IU/L penicillin G, 25 mg/L streptomycin and 10% heat-inactivated FCS. The incubation of the macrophages (10 mL in 50 mL TubeSpin^®^ bioreactors) took place on a roller culture apparatus (5 rpm) in a Heraeus Cytoperm 8080 incubator (Thermo Electron Corporation, Vienna, Austria) at 37 °C with 5% CO_2_. The cell culture was passaged two times a week.

### 4.7. Viability

#### 4.7.1. Viability of *Leishmania tarentolae* Promastigotes

Viability assays with LtP were performed with horizontal concentration gradients (three compounds were tested in duplicate at twelve different concentrations) in 96-well non-treated cell culture plates (Eppendorf, Hamburg, Germany). Assays were performed in a 1:1 mixture of yeast extract medium (YEM, 20.7 g/L yeast extract powder, 1.2 g/L K_2_HPO_4_, 0.2 g/L KH_2_PO_4_, 2.9 g/L glucose monohydrate, pH 7.4) and PBS (137 mM NaCl, 1.4 mM KH_2_PO_4_, 4.3 mM Na_2_HPO_4_ and 2.7 mM KCl, pH 7.4) supplemented with 25,000 U/L penicillin G, 25 mg/L streptomycin and 6 μM hemin and starting with 2 × 10^6^ LtP/mL. The plates were incubated for 48 h at 26 °C. Then, resazurin (20 µM) as a viability indicator was added. After another incubation of 4 h at 26 °C, the fluorescence of the plates was measured with a plate reader (Varioskan, Thermo Fisher Scientific, Waltham, MA, USA) at 560 nm excitation and 590 nm emission. For the combination viability assays with vertical concentration gradients, the radical scavengers NAC (2 mM) or the iron chelator PIH (50 µM) were added to YEM/PBS in one half of the 96-well plate while the other half contained YEM/PBS only. Otherwise, incubation and reading were done as described above. The viability (%) of LtP in each well was calculated using the quotient of the fluorescence values of the wells with LtP and compounds minus data from wells with YEM/PBS medium (without LtP) divided by fluorescence values of wells with LtP only minus fluorescence of wells with medium. From the respective viability–concentration curves, the IC_50_ values were calculated according to a four-parameter logistic model [76].

#### 4.7.2. Viability of *L. donovani* Promastigotes

The anti-promastigote activity of ErgoEP and DHCholEP was measured in log phase of LdP (MHOM/IN/1983/AG83) using a modified MTS–PMS cell viability assay [77]. In brief, log phase promastigotes (5 × 10^5^ cells/mL medium, 200 µL/well) were incubated with ErgoEP or DHCholEP (0–100 µM) for 48 h at 24 °C in M199 medium. At the end of the 48 h incubation, a 5:1 mixture of MTS (2 mg/mL) and PMS (0.92 mg/mL) was added (20 µL/well); after an incubation for 3 h at 37 °C, absorbances were measured at 492 nm. The IC_50_ values were determined by graphical extrapolation using GraphPad Prism software, version 8.4.2 (GraphPad Software Inc., La Jolla, CA, USA). The viability (%) of LdP in each well was calculated using the quotient of the absorption values of the wells with compounds minus data from wells with medium divided by absorption values of wells with cells only minus absorption of wells with medium. The IC_50_ values were obtained by graphical intrapolation of the viability–concentration curves.

#### 4.7.3. Viability of J774A.1 Macrophages

For the J774A.1 macrophages, horizontal viability assays were conducted in 96-well cell-culture-treated plates (Eppendorf, Hamburg, Germany). A cell suspension was prepared, containing DMEM, 25,000 U/L penicillin G, 25 mg/L streptomycin and 10% FCS, along with 0.2 × 10^6^ J774A.1/mL per well. After addition of the compounds in starting wells, a serial dilution generating 12 different compound concentrations was done, and the plates were incubated for 48 h at 37 °C and 5% CO_2_. Then, resazurin (20 µM) was added to the wells. After 4 h at 37 °C, the fluorescence was measured at 560 nm excitation and 590 nm emission using the Varioskan plate reader. Calculation of viability and IC_50_ values was done as described for LtP.

#### 4.7.4. Viability of Mouse Peritoneal Macrophages

The influence of ErgoEP and DHCholEP on mouse peritoneal macrophages (MPM) and the selectivity index was evaluated in resident MPM (5 × 10^5^ cells/mL medium, 200 µL/well; not elicited by thioglycolate, isolated from Swiss albino mice). MPM were allowed to adhere overnight at 37 °C, 5% CO_2_ in RPMI medium, supplemented with 10% FBS, penicillin G (50 IU/mL), streptomycin (50 μg/mL) and amphotericin B (1 µg/mL), followed by incubation with ErgoEP and DHCholEP (0–500 µM) for 48 h. The cell viability was measured by the MTS–PMS assay. In brief, a 10:1 mixture of MTS (2 mg/mL) and PMS (0.92 mg/mL) was added (20 µL) to each well and incubated for 3 h at 37 °C, and absorbances were measured at 492 nm [78]. Calculation of viability and IC_50_ values was done as described for LdP.

#### 4.7.5. Viability of *L. donovani* Amastigotes

To assess the anti-amastigote activity of ErgoEP and DHCholEP, peritoneal macrophages were ex vivo infected with LdP, and the effect of sterol EPs was studied. In brief, resident MPM were allowed to adhere overnight in RPMI medium (Phenol Red^+^) supplemented with 10% FBS, penicillin G (50 IU/mL) and streptomycin (50 μg/mL) in 12-well plates at 37 °C, 5% CO_2_. Next, infection was allowed to be established for 24 h using 5–7-day-old stationary metacyclic LdP [MHOM/IN/1983/AG83] at a ratio of 10:1 (parasite:macrophage). This was followed by addition of ErgoEP (0–25 µM) and DHCholEP (0–25 µM) for an additional 48 h at 37 °C, 5% CO_2_ [78]. 

Total RNA from infected peritoneal macrophages was extracted using the TRIzol method, with the RNA quality and quantity verified by measuring absorbances at 260/280 nm using NanoDrop^TM^ One^C^ (Thermo Scientific, Waltham, MA, USA). Following conversion of 1 µg total RNA to cDNA using a one-step cDNA reverse transcription kit, absolute quantification was performed by ddPCR using primers for amastigote-specific *A2* gene (F, 5′-CTGCAGGCTGTTGACGTTTC-3′; R, 5′-AAGGTTTGCCTCGTCACCAT-3′) [79]. 

In brief, cDNA (50 ng) was added to ddPCR EvaGreen Supermix containing 125 nM of forward and reverse primers in a final volume of 20 μL. Each reaction mixture was loaded into a sample well of an eight-well disposable cartridge (DG8), along with 70 μL of droplet generation oil. Droplets were formed using a QX200 droplet generator (BioRad, Hercules, CA, USA) and were then transferred to a 96-well PCR plate to perform PCR of 40 cycles at 95 °C for 5 min, 95 °C for 30 s, annealing 58 °C for 1 min, followed by a final extension at 90 °C for 5 min. The resultant products were scanned on a QX200 Droplet Reader (BioRad, Hercules, CA, USA). Data were analyzed using QuantaSoft software, version 1.7.4 [80], and quantified in terms of copies/µL. The IC_50_ was determined by graphical extrapolation using GraphPad Prism version 8.4.2 (GraphPad Software Inc., La Jolla, CA, USA). The primers were sourced by NCBI Primer-BLAST and confirmed by UCSC In-Silico PCR.

### 4.8. Large-Scale Incubations of LtP with Compounds

For sterol extraction experiments, BHI medium (45 mL) was distributed in each 50 mL centrifuge tube along with an aliquot of LtP culture containing 36 × 10^6^ LtP/mL. Then, targeted concentrations of the compounds MiAz, ErgoEP, DHCholEP and AA were set, and the corresponding amount of solvent (EtOH) was added in control tubes. The samples were prepared in triplicates for each compound concentration. Therefore, 15 mL aliquots of the treated cell suspensions were distributed into three 50 mL centrifuge tubes which had holes for aeration. The cells were incubated for 24 h at 26 °C in a shaker, and the cell density was measured photometrically after 0, 6 and 24 h. Finally, the percentage of LtP growth in tubes with compounds was calculated (LtP EtOH control = 100%). The individual cell batches were used for sterol extraction.

For oximetry and superoxide radical detection, in batch incubations of LtP with sterol EPs to grow sufficient cells for the experiments, the initial cell density was adjusted to 18 × 10^6^ LtP/mL in 40 mL BHI medium. Aliquots of 10 mL cell suspension were distributed into four 50 mL tubes with holes in the cap to allow oxygen supply. Then, sterol EPs were added into three of the tubes to obtain three different concentrations: 2.5 µM, 5 µM, 10 µM for ErgoEP and 1.25 µM, 2.5 µM, 5 µM for DHCholEP. For the control tube, an equivalent volume of EtOH was added corresponding to the highest sterol EP concentration. The cell suspensions were incubated for 24 h at 26 °C in a shaker. The cell density of each tube was measured at 2.5 h, 5 h and 24 h. From a total of four tubes with cells, always the LtP control sample and LtP samples treated with the respective sterol EP, which exhibited growth inhibition but still contained a sufficient number of cells (in most cases 2.5 µM ErgoEP or 1.25 µM DHCholEP), were selected for superoxide radical detection and oximetry.

#### Sterol Extraction

For sterol extraction, LtP-containing tubes from batch incubations were centrifuged at 3000× *g* and 20 °C for 10 min. Afterwards, the supernatants were removed and the pellets were resuspended in 5 mL PBS and transferred to weighed 5 mL reaction tubes. Subsequent to the centrifugation of the resuspended pellet (4000× *g*, 10 min, 20 °C) and removal of the supernatant, the weight of the LtP pellet was determined for each reaction tube. Next, 1.5 mL of the saponification solution (6.25 g of KOH, 8.75 mL of H_2_O and 16.25 mL of EtOH) was distributed to each reaction tube. Then, the tubes were incubated for 1 h at 85 °C and vortexed every 15 min. Once the tubes had cooled down to room temperature, 0.5 mL of H_2_O and 1.5 mL of n-hexane were added to each tube. After mixing on an Eppendorf thermomixer (900 rpm, 30 min, 20 °C) and centrifugation (4000× *g*, 10 min, 20 °C), the organic phase (1.2 mL) was transferred to an empty 1.5 mL Eppendorf tube. The organic layers were evaporated under a stream of N_2_. The dried extracts were dissolved in ACN (1.2 mL), and the solution was transferred to HPLC vials.

### 4.9. High-Performance Liquid Chromatography

HPLC analysis was conducted on an LC-20 system (Shimadzu, Kyoto, Japan) with an included dual-wavelength UV detector and the LCSolutions 2.1 software. A volume of 10 μL of each sample was injected by an autosampler on an RP-8 column (LiChrospher^®^, analytical grade RP-8, particle size 5 μm, ID 4 mm, length 250 mm) with a pre-column (LiChrospher^®^, RP-8, particle size 5 μm, ID 4 mm, length 4 mm). Samples were eluted with 10% H_2_O and 90% ACN/MeOH (99:1, *v*/*v*) for 45 min, and detection was performed by UV detection at a 205 nm and a 281 nm channel. For quantification, the peak areas were measured using the LCSolutions software.

### 4.10. Detection of Superoxide Radicals

For the detection of superoxide radicals in LtP cultures, EPR measurements with CMH were conducted on LtP incubated with sterol EPs for 20 min or 24 h. An aliquot of the respective LtP culture corresponding to 375 × 10^6^ LtP was transferred into a 1.5 mL Eppendorf tube and centrifuged (Centrifuge Z 366 K, Hermle, Gosheim, Germany) at 20 °C and 2000× *g* for 10 min. The supernatant was discarded, and the cell pellet was resuspended in 1.5 mL of PBS and centrifuged again. After the second centrifugation and removal of the supernatant, the pellet was again resuspended in 1.5 mL of PBS. Aliquots of 100 µL (250 × 10^6^ LtP/mL) were transferred into 1.5 mL Eppendorf tubes and incubated on a thermomixer for 20 min at 26 °C with respective compounds. Tubes with 100 µL of PBS were used as negative controls. 

For each experimental day, four replicates per sample type were measured. For the short-term incubations (20 min), the replicates included an LtP suspension sample without antileishmanial compounds, one incubated for 5 min with AA (0.36 µM) and one incubated for 20 min with ErgoEP (362 µM) or DHCholEP (362 µM). For the long-term (24 h) incubation, the replicates included a control LtP suspension sample without sterol EPs, a control LtP suspension incubated for 5 min with AA (0.36 µM), LtP incubated for 24 h with 2.5 µM ErgoEP or 1.25 µM DHCholEP and a sterol-EP-incubated LtP suspension with added AA (0.36 µM, 5 min) (final concentrations given). 

Finally, 6.5 µL premix containing deferoxamine (DFO, 92 µM), diethylenetriaminepentaacetic acid (DTPA, 23 µM) and glucose monohydrate (11.2 mM) was added to each sample tube, and, after 2 min incubation, CMH (369 µM) was added to the respective sample directly before the measurement (final concentrations given). An aliquot was aspirated into a 50 µL capillary, sealed and transferred to the MD5 resonator of the EPR spectrometer (EMX Digital Upgrade, Bruker, Rheinstetten, Germany). The measurement was begun using the following parameters: microwave frequency 9.6839 GHz; microwave power 20 mW; modulation frequency 100 kHz; modulation amplitude 1 G; center field 3448 G; sweep width 100 G; attenuation 70 dB; 5 consecutive scans; measurement temperature 25 °C. From the recorded EPR spectra, the peak-to-peak intensities of the respective mid-field peak of the CM^●^ were measured at 5 time points, and the relative rate of CMH oxidation in peak-to-peak intensity/min was calculated.

### 4.11. Oximetry

To assay the effect of compounds on oxygen consumption of LtP, a Clark-type oxygen electrode (Hansatech Instruments, Norfolk, UK) and the software MCREC 1.51 were used. LtP at 10^8^ cells/mL in BHI medium (25 °C) were added and treated with increasing concentrations of test compounds. Each concentration was assayed in quadruplicates, and the results were expressed as percentage of oxygen consumption in comparison with the untreated control LtP. The highest concentration of the respective vehicles caused only a 2% inhibition.

To assess further mitochondrial bioenergetic parameters of LtP, Oxoplates with a round bottom (Type OP96U, PreSens Precision Sensing, Regensburg, Germany) with integrated chemical optical oxygen sensors were used. The oxygen sensors are composed of an indicator and reference layer. Prior to kinetic measurements, the Oxoplates were calibrated once per plate, performing a two-point calibration with air-saturated BHI medium and oxygen-free BHI medium. The BHI medium was air-saturated by agitating with a magnetic stirrer in an open beaker for at least 1 h and deoxygenated by adding sodium dithionite (50 mg/mL). Then, 200 µL of the air-saturated medium was dispensed into row A (column 1–6) and 200 µL of the deoxygenated medium into row A (column 7–12). The wells were then quickly sealed with 65 µL paraffin oil per well. The fluorescence was measured at 540 nm excitation and 650 nm emission for the oxygen indicator layer and at 540 nm excitation and 590 nm emission for the reference layer with the Varioskan plate reader. For kinetic measurements, the wells in row B were loaded with 200 µL air-saturated BHI medium to compensate for temperature drifts. Row C was loaded with 50 µL BHI medium only. Rows D–H were filled with 50 µL BHI medium containing different mitochondrial inhibitors/uncouplers (final concentrations): 5 µM oligomycin (row D–G), and additionally 0.5 µM CCCP (row E), 1 µM CCCP (row F), 2 µM CCCP (row G) and 2 µM AA plus 2 µM Rot (row H), respectively. 

For short-term incubations with sterol EPs, an aliquot of LtP cell culture was centrifuged in a 50 mL tube for 10 min at 2000× *g* and 20 °C. The cell pellet was resuspended in air-saturated BHI medium, aiming at a cell density of 150 × 10^6^ LtP/mL. This LtP suspension was split in two equal aliquots and supplemented with either 362 µM ErgoEP or 362 µM DHCholEP (final concentrations). Then, 150 µL of these cell suspensions was immediately added to the wells in rows C–H (ErgoEP-treated LtP in columns 1–6; DHCholEP-treated LtP in columns 7–12). For long-term incubations with sterol EPs, the respective LtP aliquots, incubated for 24 h with 2.5 µM ErgoEP or 1.25 µM DHCholEP, were centrifuged and resuspended in separate 50 mL tubes (150 × 10^6^ LtP/mL) and loaded on the Oxoplates, prepared as described above.

Finally, the wells were quickly sealed with 65 µL of paraffin oil, the plate was transferred to the plate reader and the kinetic measurements were begun. The fully loaded and sealed Oxoplates were measured at the mentioned fluorescence wavelength pairs 14 times at intervals of 3 min, resulting in a total measurement time of about 40 min. Oxygen consumption values were calculated from fluorescence intensities in a customized Excel worksheet, following the calibration procedure listed in the manufacturer’s manual. The linear slopes of the oxygen concentration decay (before anaerobiosis) were used to calculate the oxygen consumption rates in µM O_2_/min.

### 4.12. Statistical Analysis

Each experiment was performed at least three times in duplicates. Results were expressed as mean ± SE or SD (as indicated). Statistical analysis was evaluated by the Kruskal–Wallis multiple comparison test followed by Dunnett’s multiple comparison test for non-parametric data using GraphPad Prism, version 8.4.2 (GraphPad Software Inc., La Jolla, CA, USA); *p* < 0.05 was considered as statistically significant.

## 5. Conclusions

Sterol EPs react with low-molecular-weight Fe^2+^ to generate both carbon-centered and oxygen-centered radicals. Unlike other antileishmanial EPs, however, this radical-producing capacity appears to contribute only marginally to the activity of sterol EPs against *Leishmania*. This limited role likely reflects the high lipophilicity of sterol EPs, which drives their activation in tight association with redox-active proteins, as for example heme-containing enzymes, and in membranes. In such a bound state, radical formation cannot be intercepted by small-molecule scavengers or iron chelators. In LtP, sterol EPs induce only minor increases in superoxide production, pointing instead to effects mediated by more selective biochemical interactions. Similarly, sterol EPs cause a slight reduction in mitochondrial respiration in LtP while paradoxically improving overall respiratory efficiency—evidence that mitochondria are not the primary target of these compounds. Taken together, these observations reinforce the view that inhibition of *Leishmania* sterol biosynthesis by sterol EPs is the dominant mechanism underlying their antileishmanial efficacy, rather than free-radical generation or mitochondrial impairment.

## Figures and Tables

**Table 1:** Formation rates of the XO/Fe^3+^ complex. From the increase of absorption at 560 nm over time, the initial slopes were calculated. MeOH/H_2_O (9:1 *v*/*v*) was used as photometric reference. Sterol stocks in EtOH were used, and therefore EtOH as vehicle was included as an additional sample. Data represent mean ± SD of three experiments. * Indicates statistically significant differences vs. EtOH vehicle (*p* < 0.05).

Compound	XO/Fe^3+^ Formation Rate (nmol/min/mL)
EtOH	0.03 ± 0.00
Chol	0.03 ± 0.01
Ergo	9.61 ± 1.38 *
ErgoEP	7.24 ± 0.19 *
DHChol	14.00 ± 0.40 *
DHCholEP	8.55 ± 0.97 *

**Table 2:** Parameters used for the simulation of EPR spectra obtained during the reaction of sterols and sterol EPs with Fe^2+^ using DMPO as spin trap compound. Simulated spectral parameters and tentative assignments are given. Below each compound, the contribution of different spin adducts with their nitrogen- (a_N_) and hydrogen- (a_ßH_, a_γH_) coupling constants and their ratio (a_N_/a_H_) are listed. More simulation parameters and the simulation output are listed in Table S4 and Figures S15–S18 in the Supplementary Information.

Compound, Species No.	Spin Adduct					
	Contrib. (%)	a_N_ (G)	a_ßH_ (G)	a_γH_ (G)	a_N_/a_H_	Assignment
ErgoEP						
1	63	14.2	9.5	1.6	1.49	RO^●^
2	19	14.7	18.1	-	0.81	-
3	18	15.5	22.7	-	0.68	RC^●^
DHCholEP						
1	38	16.0	22.1	-	0.72	RC^●^
2	27	16.5	14.8	-	1.11	ROO^●^
3	21	13.9	9.6	1.4	1.45	RO^●^
4	12	15.0	12.1	-	1.23	ROO^●^
Ergo						
1	45	14.0	9.4	1.5	1.48	RO^●^
2	36	14.6	13.5	-	1.08	ROO^●^
3	17	15.5	23.0	-	0.67	RC^●^
4	2	16.3	15.0	-	1.09	ROO^●^
DHChol						
1	64	14.6	13.6	-	1.07	ROO^●^
2	24	13.8	9.1	1.3	1.51	RO^●^
3	10	15.5	22.6	-	0.68	RC^●^
4	2	14.9	18.0	-	0.83	-

**Table 3:** Influence of sterols and sterol EPs on the viability of *Leishmania donovani* promastigotes (LdP) and murine peritoneal macrophages (MPM). IC_50_ values represent mean ± SEM (*n* = 3) of at least three experiments in duplicates or the highest concentration tested in the respective cells. The selectivity index was calculated as the ratio of IC_50_ values of MPM to LdP.

Compound	IC_50_ (µM)		Selectivity Index
	LdP	MPM	
Ergo	58.8 ± 14.0	>500	>8
ErgoEP	18.3 ± 7.4	>500	>27
DHChol	37.8 ± 9.8	>500	>13
DHCholEP	8.3 ± 2.1	>500	>60

**Table 4:** Influence of sterols, sterol EPs and related compounds on the viability of *Leishmania tarentolae* promastigotes (LtP) and J774A.1 macrophages. IC_50_ values represent mean ± SD of five to seven cell culture batches; selectivity index was calculated as the ratio of IC_50_ values of J774A.1 to LtP. Related compounds tested were pentamidine (Pen), antimycin A (AA), miltefosine (Mil), amphotericin B (AmpB) and miconazole (MiAz). * Data for artemisinin (Art) originating from [16] are shown for comparison.

Compound	IC_50_ (µM)		Selectivity Index
	LtP	J774A.1	
Pen	0.790 ± 0.132	1.22 ± 0.79	2
Ergo	18 ± 14	22 ± 12	1
ErgoEP	4.5 ± 1.2	22 ± 5	5
DHChol	5.2 ± 4.7	17 ± 6	3
DHCholEP	2.0 ± 0.4	15 ± 5	8
Mil	0.267 ± 0.147	124 ± 43	464
AmpB	0.0187 ± 0.0085	32 ± 13	248
MiAz	0.998 ± 0.199	26 ± 12	26
Art	2.96 ± 0.71 *	192 ± 41 *	65
AA	0.0025 ± 0.0022	36.2 ± 12.2	14,480

**Table 5:** Relative change in IC_50_ values of amphotericin B (AmpB), artemisinin (Art), ergosterol endoperoxide (ErgoEP) and dehydrocholesterol endoperoxide (DHCholEP) in LtP in the presence and absence (100%) of 2 mM N-acetylcysteine (NAC) or of 50 µM pyridoxal isonicotinoyl hydrazone (PIH). Data represent mean ± SD of three experiments.

Compound	Relative Change of IC_50_ (%)	
	with NAC	with PIH
ErgoEP	124 ± 20	69.7 ± 19.7
DHCholEP	93 ± 4	92.6 ± 23.4
AmpB	554 ± 80	-
Art	-	180.5 ± 47.3

**Table 6:** Bioenergetic parameters of LtP and LtP short-term-treated with ErgoEP (362 µM, 40 min) and long-term-treated with ErgoEP (2.5 µM, 24 h). The data show the mean value from six cell batches (*n* = 6) ± SD. * Indicates statistically significant differences vs. untreated LtP group (*p* < 0.05).

		Short		Long	
Parameter	Unit	LtP	LtP + ErgoEP	LtP	LtP + ErgoEP
Coupling Efficiency		0.22 ± 0.07	0.19 ± 0.09	0.28 ± 0.05	0.36 ± 0.08 *
Respiratory Control Ratio		1.55 ± 0.27	1.56 ± 0.08	1.65 ± 0.035	2.17 ± 0.57
Spare Resp Capacity	pmol/min/10^6^ LtP	16.9 ± 17.6	18.6 ± 6.5	16.1 ± 16.7	24.6 ± 15.5
Non-mitochondrial Resp	pmol/min/10^6^ LtP	5.80 ± 2.97	6.94 ± 1.90	6.05 ± 3.08	4.48 ± 3.66
Mitochondrial Resp	pmol/min/10^6^ LtP	93.6 ± 13.0	76.5 ± 11.6 *	90.6 ± 6.2	69.3 ± 10.6 *
Proton Leak	pmol/min/10^6^ LtP	73.0 ± 13.4	60.8 ± 4.1	65.8 ± 8.4	44.5 ± 11.1 *
ATP Turnover	pmol/min/10^6^ LtP	20.6 ± 6.7	15.6 ± 8.3	24.7 ± 3.4	24.8 ± 4.4
Max Mito Resp	pmol/min/10^6^ LtP	110.6 ± 13.9	95.1 ± 9.5 *	106.7 ± 14.6	94.0 ± 21.5
Proton Leak (Relative)	% of Mito Resp	77.8 ± 6.7	80.5 ± 8.5	72.4 ± 4.9	63.6 ± 7.6 *
ATP Turnover (Relative)	% of Mito Resp	22.1 ± 6.7	19.4 ± 8.5	27.5 ± 4.9	36.3 ± 7.6 *
Max Mito Resp (Relative)	% of Mito Resp	119.6 ± 19.2	125.4 ± 11.0	118.4 ± 18.9	135.0 ± 22.9

**Table 7:** Bioenergetic parameters of LtP and LtP short-term-treated with DHCholEP (362 µM, 40 min) and long-term-treated with DHCholEP (1.25 µM, 24 h). The data show the mean value from six cell batches (*n* = 6) ± SD. * Indicates statistically significant differences vs. untreated LtP group (*p* < 0.05).

		Short		Long	
Parameter	Unit	LtP	LtP + DHCholEP	LtP	LtP + DHCholEP
Coupling Efficiency		0.17 ± 0.06	0.15 ± 0.08	0.23 ± 0.05	0.36 ± 0.07 *
Respiratory Control Ratio		1.56 ± 0.20	1.44 ± 0.09	1.58 ± 0.24	2.06 ± 0.45 *
Spare Resp Capacity	pmol/min/10^6^ LtP	25.1 ± 11.0	15.1 ± 8.5	19.3 ± 13.8	23.2 ± 18.4
Non-mitochondrial Resp	pmol/min/10^6^ LtP	5.08 ± 3.71	5.37 ± 2.91	6.51 ± 3.23	6.48 ± 2.55
Mitochondrial Resp	pmol/min/10^6^ LtP	89.7 ± 8.7	71.5 ± 16.0 *	92.5 ± 6.5	77.9 ± 11.3 *
Proton Leak	pmol/min/10^6^ LtP	74.0 ± 8.5	60.3 ± 10.4 *	71.1 ± 7.5	50.1 ± 10.0 *
ATP Turnover	pmol/min/10^6^ LtP	15.6 ± 5.7	11.1 ± 8.4	21.3 ± 3.6	27.8 ± 5.8 *
Max Mito Resp	pmol/min/10^6^ LtP	114.8 ± 16.5	86.6 ± 12.5 *	111.8 ± 16.6	101.2 ± 18.7
Proton Leak (Relative)	% of Mito Resp	82.6 ± 5.9	85.2 ± 7.7	76.8 ± 4.6	64.2 ± 7.0 *
ATP Turnover (Relative)	% of Mito Resp	17.4 ± 5.9	14.7 ± 7.7	23.1 ± 4.6	35.8 ± 7.0 *
Max Mito Resp (Relative)	% of Mito Resp	127.8 ± 11.4	123.0 ± 13.3	120.8 ± 14.5	131.3 ± 26.1

**Table 8:** Elution of sterols and related compounds in reversed-phase HPLC analysis. Retention times (RT) and ratios of peak areas at the retention time in the 281 nm and 205 nm UV detection channels were measured. Chromatograms were obtained from pure standard compounds or NMR-characterized fractions. Compounds were dehydrocholesterol endoperoxide (DHCholEP), ergosterol endoperoxide (ErgoEP), 5-dehydroepisterol (5-DHE), 7-dehydrocholesterol (DHChol), ergosterol (Ergo) and cholesterol (Chol).

Compound	RT (min)	Ratio Area 281 nm/Area 205 nm
DHCholEP	6.9	0.016
ErgoEP	7.1	0.007
5-DHE	12.2	5.864
DHChol	14.3	3.450
Ergo	14.6	1.971
Chol	18.5	0.000

## Data Availability

Data are available from the corresponding author upon request.

## Supplementary Materials

The following supporting information can be downloaded at: https://www.mdpi.com/article/10.3390/molecules31060979/s1, Figure S1. Structure and carbon atom numbering for ErgoEP; Figure S2. Evaluation of anti-amastigote efficacy of sterol EPs, Table S1. ^1^H NMR shifts of ergosterol derivatives recorded in CDCl_3_, Table S2. ^1^H NMR shift of DHCHolEP recorded in CDCl_3_, Table S3. ^13^C-NMR shifts of sterol derivatives recorded in CDCl_3_; Figure S3. ^1^H NMR of DHCholEP, Figure S4. ^13^C NMR of DHCholEP; Figure S5. COSY of DHCholEP, Figure S6. edited gs-HSQC of DHCholEP; Figure S7. gs-HMBC of DHCholEP; Figure S8. gs-NOESY of DHCholEP; Figure S9. ^1^H NMR of mixture of ErgoEP and DHErgolEP; Figure S10. ^13^C NMR of mixture of ErgoEP and DHErgolEP; Figure S11. COSY of mixture of ErgoEP and DHErgolEP; Figure S12. edited gs-HSQC of mixture of ErgoEP and DHErgolEP; Figure S13. gs-HMBC of mixture of ErgoEP and DHErgolEP; Figure S14. gs-NOESY of mixture of ErgoEP and DHErgolEP; Table S4. Extended parameters used for the simulation in the program WINSIM 2002; Figure S15. Simulation of DMPO spin adducts obtained from the reaction of ErgoEP with Fe^2+^; Figure S16. Simulation of DMPO spin adducts obtained from the reaction of DHCholEP with Fe^2+^; Figure S17. Simulation of DMPO spin adducts obtained from the reaction of Ergo with Fe^2+^; Figure S18. Simulation of DMPO spin adducts obtained from the reaction of DHChol with Fe^2+^.

## Abbreviations

The following abbreviations are used in this manuscript:

5-DHE	5-dehydroepisterol
AA	antimycin A
ACN	acetonitrile
ATP	adenosine triphosphate
AmpB	amphotericin B
Art	artemisinin
BHI	brain heart infusion
CCCP	carbonyl cyanide 3-chlorophenylhydrazone
Chol	cholesterol
CMH	1-hydroxy-3-methoxycarbonyl-2,2,5,5-tetramethylpyrrolidine
CM^●^	1-oxyl-3-methoxycarbonyl-2,2,5,5-tetramethylpyrrolidine
DFO	deferoxamine
DHChol	7-dehydrocholesterol
DHCholEP	dehydrocholesterol endoperoxide
DHErgoEP	dehydroergosterol endoperoxide
DMEM	Dulbecco’s modified Eagle’s medium
DMPO	5,5-dimethyl-1-pyrroline-N-oxide
DMSO	dimethyl sulfoxide
DTPA	diethylenetriaminepentaacetic acid
EP	endoperoxide
EPR	electron paramagnetic resonance
Ergo	ergosterol
ErgoEP	ergosterol endoperoxide
EtOH	ethanol
FBS	fetal bovine serum
FCS	fetal calf serum
IC_50_	half-maximal inhibitory concentration
LdP	*Leishmania donovani* promastigotes
LtP	*Leishmania tarentolae* promastigotes
MB	methylene blue
MeOH	methanol
MiAz	miconazole
Mil	miltefosine
MPM	mouse peritoneal macrophages
MTS	3-(4,5-dimethylthiazol-2-yl)-5-(3-carboxymethoxyphenyl)-2-(4-sulfophenyl)-2H-tetrazolium
NAC	N-acetylcysteine
NMR	nuclear magnetic resonance
OCR	oxygen consumption rate
OD	optical density
Oligo	oligomycin
PBS	phosphate-buffered saline
PE	petrol ether
Pen	pentamidine
PIH	pyridoxal isonicotinoyl hydrazone
PMS	phenazine methosulfate
Rot	rotenone
RPMI	Roswell Park Memorial Institute
STAT	signal transducer and activator of transcription
TDHCholEP	tetradehydrocholesterol endoperoxide
TLC	thin-layer chromatography
VLC	vacuum liquid chromatography
XO	xylenol orange
YEM	yeast extract medium
