# Supplementary material for: Sterol Endoperoxides and Their Antileishmanial Effects: Influence on Viability, Oxygen Metabolism and Sterol Synthesis
Source: Molecules. 2026 Mar 14;31(6):979. doi: 10.3390/molecules31060979 (PMC13029385; doi:10.3390/molecules31060979)
Supplement: Supplementary file 1 [file molecules-31-00979-s001.zip › molecules-4137085-supplementary.pdf]

Sterol Endoperoxides and their Antileishmanial Effects: Influence on Viability, Oxygen Metabolism and Sterol Synthesis

Sterol Endoperoxides in *Leishmania*

D. Sarkar[1,2], A. Aleta[1], M. Ahmetašević[1], M. Tosin[1], L. Machin[1,3], E. Schrödl[1], M. Bacher[4], T. Rosenau[4], L. Monzote[5], K. Staniek[1], M. Chatterjee[2], L. Gille[1]

[1] Pharmacology and Toxicology, Department of Biological Sciences and Pathobiology, University of Veterinary Medicine, Vienna, Austria

[2] Department of Pharmacology, Institute of Postgraduate Medical Education & Research, Kolkata, India

[3] Pharmacy Department, Institute of Pharmacy and Food Sciences, University of Havana, Cuba

[4] Institute of Chemistry of Renewable Resources, Department of Natural Sciences and Sustainable Resources, BOKU University, UFT Research Center, Tulln, Austria

[5] Parasitology Department, Institute of Tropical Medicine “Pedro Kouri”, Havana, Cuba

Supplementary information

Supplementary information

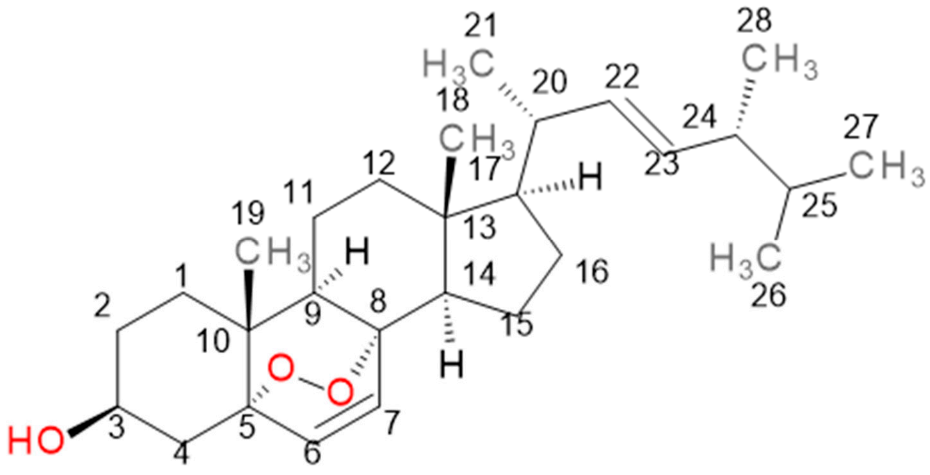

Figure S1. Structure and carbon atom numbering for ErgoEP.

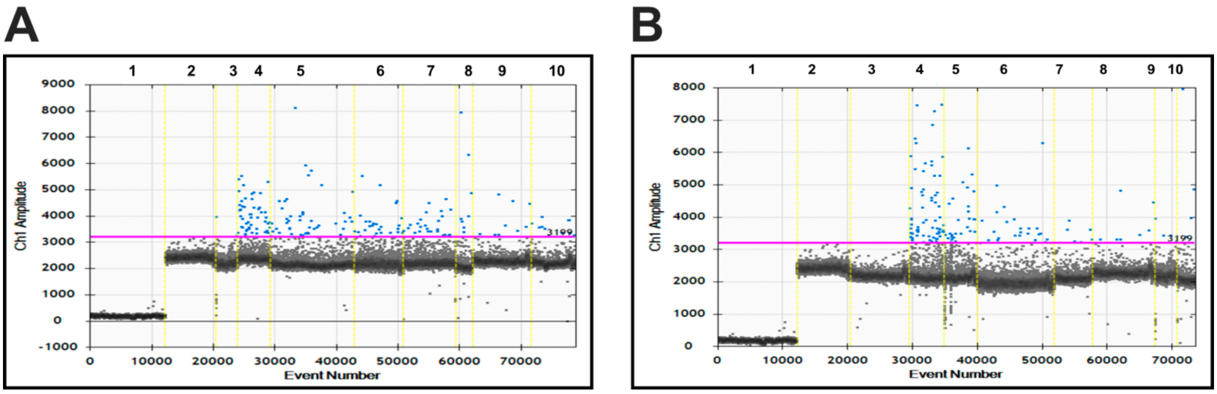

Figure S2. Evaluation of anti-amastigote efficacy of sterol EPs. Representative profiles of absolute quantification of amastigote-specific *A2* gene by droplet digital PCR (A, B) in uninfected and *Leishmania donovani*-infected mouse peritoneal macrophages (MPM). In 1-D plots of amplitude of fluorescence signals emitted from droplets, lane 1 indicates negative control (nuclease-free water), lane 2 – non-template control, lane 3 – uninfected peritoneal macrophages, lane 4 – infected peritoneal macrophages, and lanes 5 - 10 – infected macrophages treated for 48 h with ErgoEP (0.78 - 25 μM; A) and DHCholEP (0.78 - 25 μM; C). EvaGreen-bound *A2*- positive droplets are shown in blue while negative droplets are shown in black.

Table S1. ^{1}H NMR shifts of ergosterol derivatives recorded in $CDCl_3$.

position	Ergo	ErgoEP	DHErgoEP
1	1.89 + 1.30 (m, each 1H)	1.95 + 1.69 (m, each 1H)	2.06 + 1.67 (m, each 1H)
2	1.89 + 1.49 (m, each 1H)	1.84 + 1.53 (m, each 1H)	1.91 + 1.56 (m, each 1H)
3	3.64 (m, 1H)	3.96 (1H, m)	4.00 (1H, m)
4	2.47 (1H, ddd, *J* = 14.3, 4.7, 2.1)<br>2.28 (1H, br.t, *J* = 13.1)	2.11 + 1.91 (m, each 1H)	2.11 + 1.91 (m, each 1H)
5	-	-	-
6	5.57 (1H, dd, *J* = 5.6, 2.2)	6.24 (d, 1H, *J* = 8.6)	6.28 (d, 1H, *J* = 8.6)
7	5.38 (m, 1H)	6.50 (d, 1H, *J* = 8.6)	6.59 (d, 1H, *J* = 8.6)
8	-	-	-
9	1.97 (m, 1H)	1.49 (m, 1H)	-
10	-	-	-
11	1.72 + 1.59 (each m, 1H)	1.50 + 1.20 (m, each 1H)	5.42 (dd, 1H, *J* = 6.0, 2.0)
12	2.06 + 1.25 (each m, 1H)	1.95 + 1.23 (m, each 1H)	2.26 (dd, 1H, J = 17.0, 6.1)<br>2.07 (m, 1H)
13	-	-	-
14	1.89 (m, 1H)	1.55 (m, 1H)	1.83 (m, 1H)
15	1.65 + 1.35 (each m, 1H)	1.59 + 1.41 (m, each 1H)	1.67 + 1.60 (m, each 1H)
16	1.75 + 1.31 (each m, 1H)	1.76 + 1.34 (m, each 1H)	1.76 + 1.34 (m, each 1H)
17	1.25 (m, 1H)	1.21 (m, 1H)	1.35 (m, 1H)
18	0.63 (s, 3H)	0.81 (s, 3H)	0.73 (s, 3H)
19	0.94 (s, 3H)	0.88 (s, 3H)	1.08 (s, 3H)
20	2.04 (m, 1H)	2.01 (m, 1H)	2.01 (m, 1H)
21	1.03 (d, 1H, *J* = 6.7)	0.99 (d, 3H, *J* = 6.6)	1.00 (d, 3H, *J* = 6.6)
22	5.16 (dd, 1H, *J* = 15.1, 7.7)	5.13 (dd, *J* = 15.3, 8.2)	5.13 (dd, *J* = 15.3, 8.2)
23	5.22 (dd, 1H, *J* = 15.1, 7.0)	5.22 (dd, *J* = 15.3, 7.4)	5.22 (dd, *J* = 15.3, 7.4)
24	1.25 (m, 1H)	1.85 (m, 1H)	1.85 (m, 1H)
25	1.47 (m, 1H)	1.46 (m, 1H)	1.46 (m, 1H)
26	0.84* (d, 3H, *J* = 6.5)	0.83* (d, 3H, *J* = 6.6)	0.83* (d, 3H, *J* = 6.6)
27	0.82* (d, 3H, *J* = 6.5)	0.81* (d, 3H, *J* = 6.6)	0.81* (d, 3H, *J* = 6.6)
28	0.91 (d, 3H, *J* = 6.8)	0.90 (d, 3H, *J* = 6.8)	0.91 (d, 3H, *J* = 6.8)

* Assignment exchangeable within the column.

Table S2. ^{1}H NMR shift of DHCHolEP recorded in $CDCl_3$.

position	DHCholEP
1	1.94 + 1.68 (m, each 1H)
2	1.83 + 1.52 (m, each 1H)
3	3.95 (1H, m)
4	2.10 (ddd, 1H, *J* = 13.8, 4.5, 2.1)<br>1.90 (dd, 1H, *J* = 13.8, 11.8)
5	-
6	6.23 (d, 1H, *J* = 8.5)
7	6.49 (d, 1H, *J* = 8.5)
8	-
9	1.48 (m, 1H)
10	-
11	1.48 + 1.20 (m, each 1H)
12	1.97 + 1.21 (m, each 1H)
13	-
14	1.54 (m, 1H)
15	1.59 + 1.32 (m, each 1H)
16	1.90 + 1.35 (m, each 1H)
17	1.16 (m, 1H)
18	0.79 (s, 3H)
19	0.87 (s, 3H)
20	1.36 (m, 1H)
21	0.89 (d, 3H, *J* = 6.6)
22	1.32 + 0.99 (m, each 1H)
23	1.32 + 1.13 (m, each 1H)
24	1.11 (m, 1H)
25	1.50 (m, 1H)
26	0.86* (d, 3H, *J* = 6.6)
27	0.85* (d, 3H, *J* = 6.6)
28	-

* Assignment exchangeable within the column.

Table S3. ^{13}C-NMR shifts of sterol derivatives recorded in $CDCl_3$.

position	Ergo	ErgoEP	DHErgoEP	DHCholEP
1	38.35	34.65	32.53	34.65
2	31.96	29.68	30.54	30.04
3	70.44	66.41	66.28	66.38
4	40.76	36.87	36.02	36.86
5	139.76	82.14	82.69	82.14
6	135.55	135.39	135.43	135.37
7	131.94	130.70	130.70	130.71
8	141.37	79.40	78.33	79.43
9	46.21	51.03	142.48	51.01
10	37.00	36.92	37.92	36.90
11	21.08	23.36	119.71	23.37
12	39.05	39.29	41.13	39.38
13	42.79	44.52	43.57	44.69
14	54.53	51.64	48.12	51.53
15	22.98	20.59	20.85	20.57
16	28.29	28.62	28.62	28.20
17	55.68	56.14	55.81	56.37
18	12.03	12.84	12.93	12.58
19	16.26	18.15	25.52	18.12
20	40.43	39.71	39.88	35.18
21	21.06	20.85	20.68	18.53
22	119.56	135.17	135.09	35.90
23	116.25	132.26	132.38	23.75
24	42.79	42.73	42.73	39.38
25	33.06	32.02	32.02	27.94
26	19.94*	19.92*	19.92*	22.77
27	19.63*	19.61*	19.61*	22.50
28	17.59	17.53	17.53	-

* Assignment exchangeable within the column. The additional double bond in DHErgoEP vs. ErgoEP is located between atom 9 and 11 in this numbering scheme.

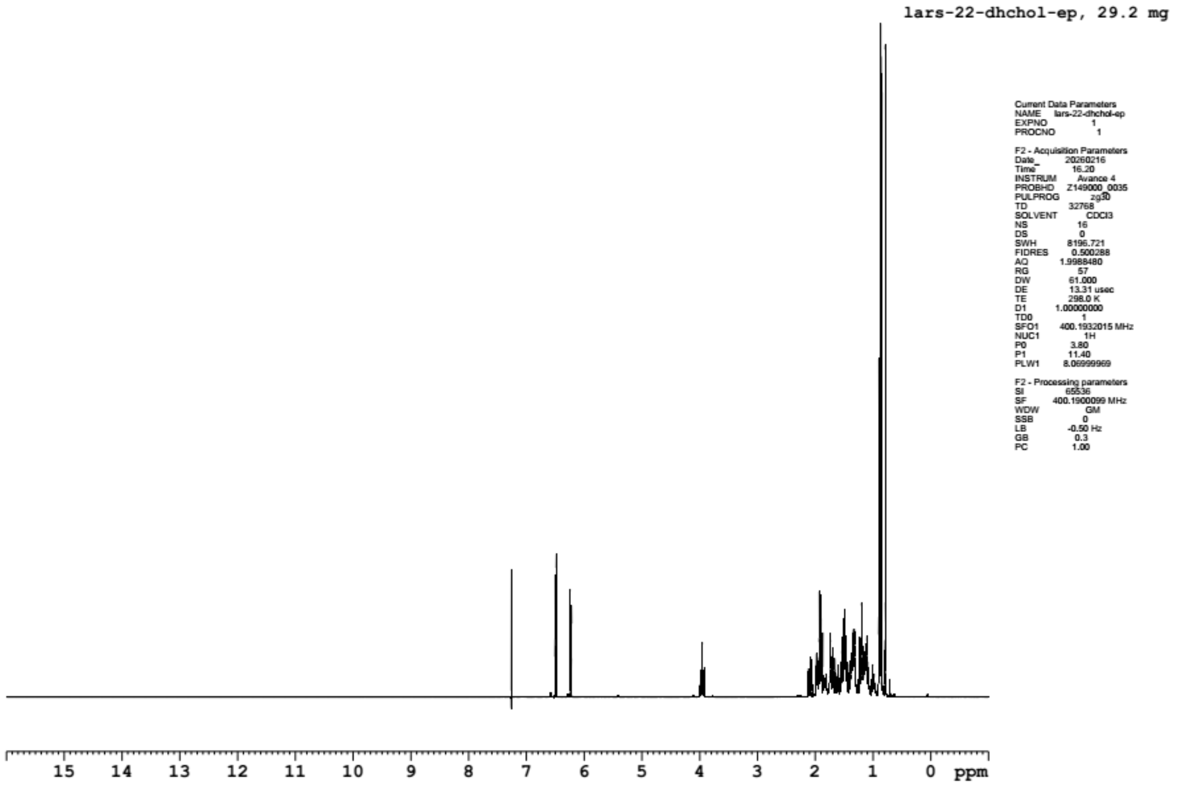

Figure S3. ^{1}H NMR of DHCholEP.

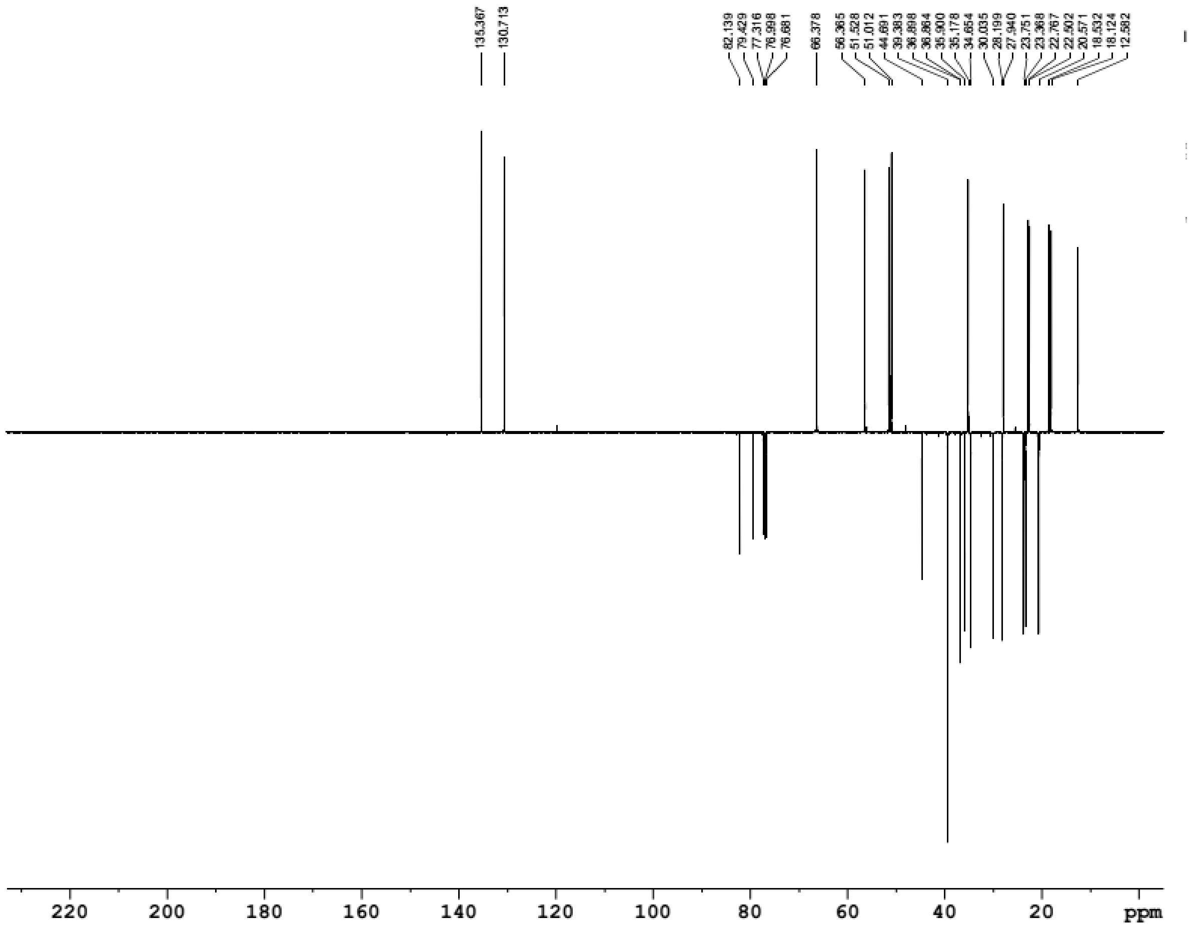

Figure S4. ^{13}C NMR of DHCholEP.

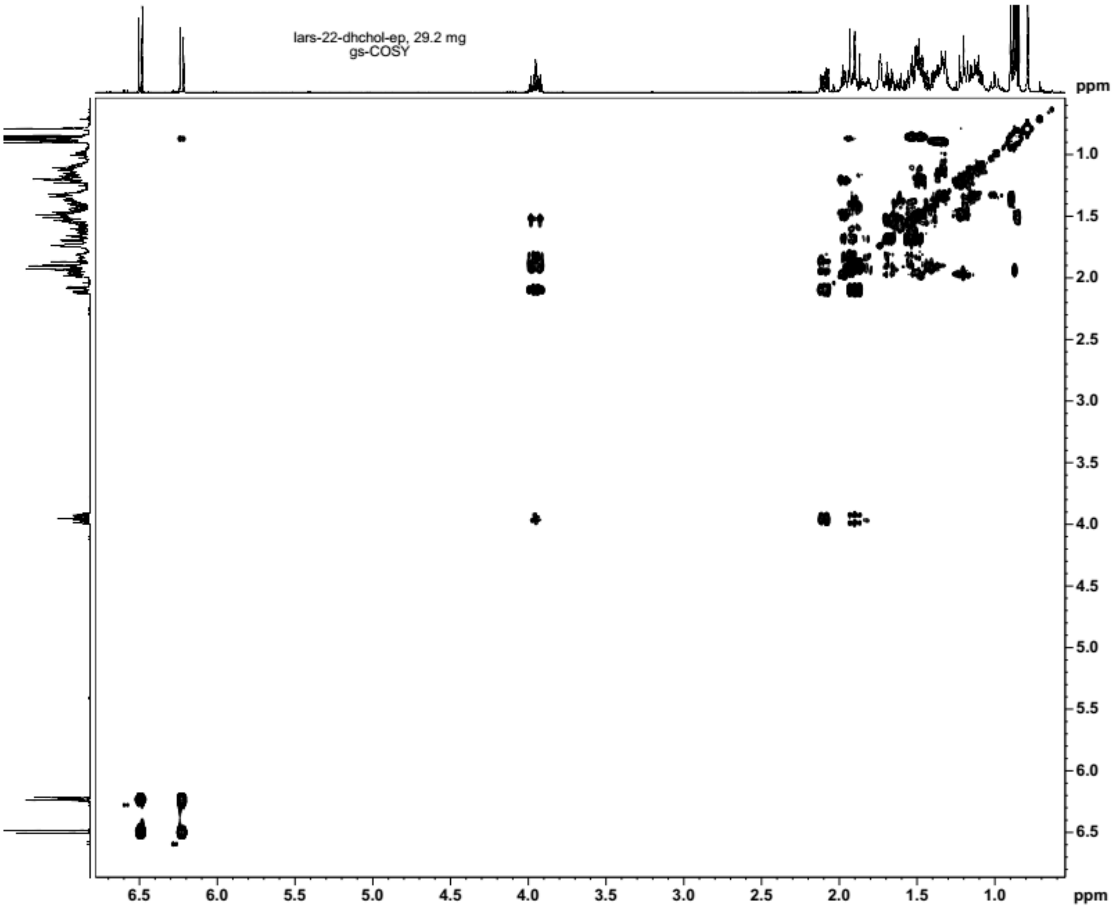

Figure S5. COSY of DHCholEP.

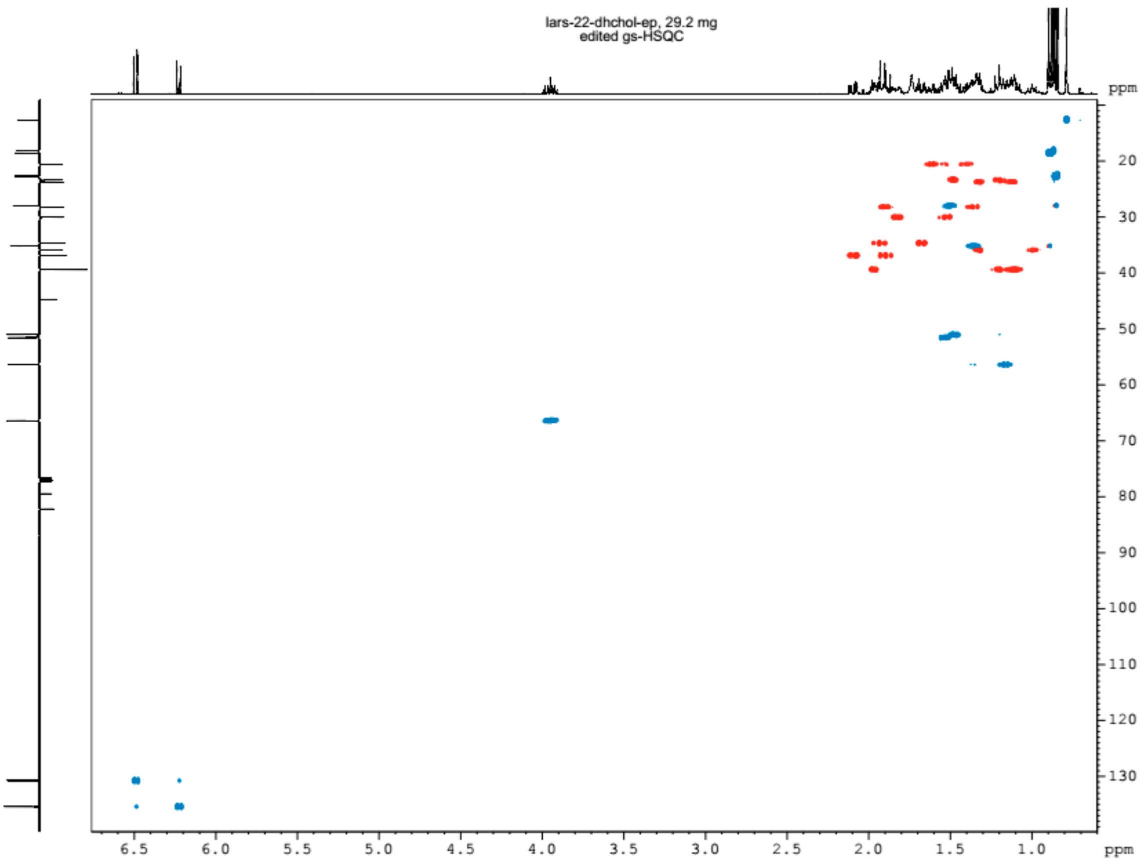

Figure S6. edited gs-HSQC of DHCholEP.

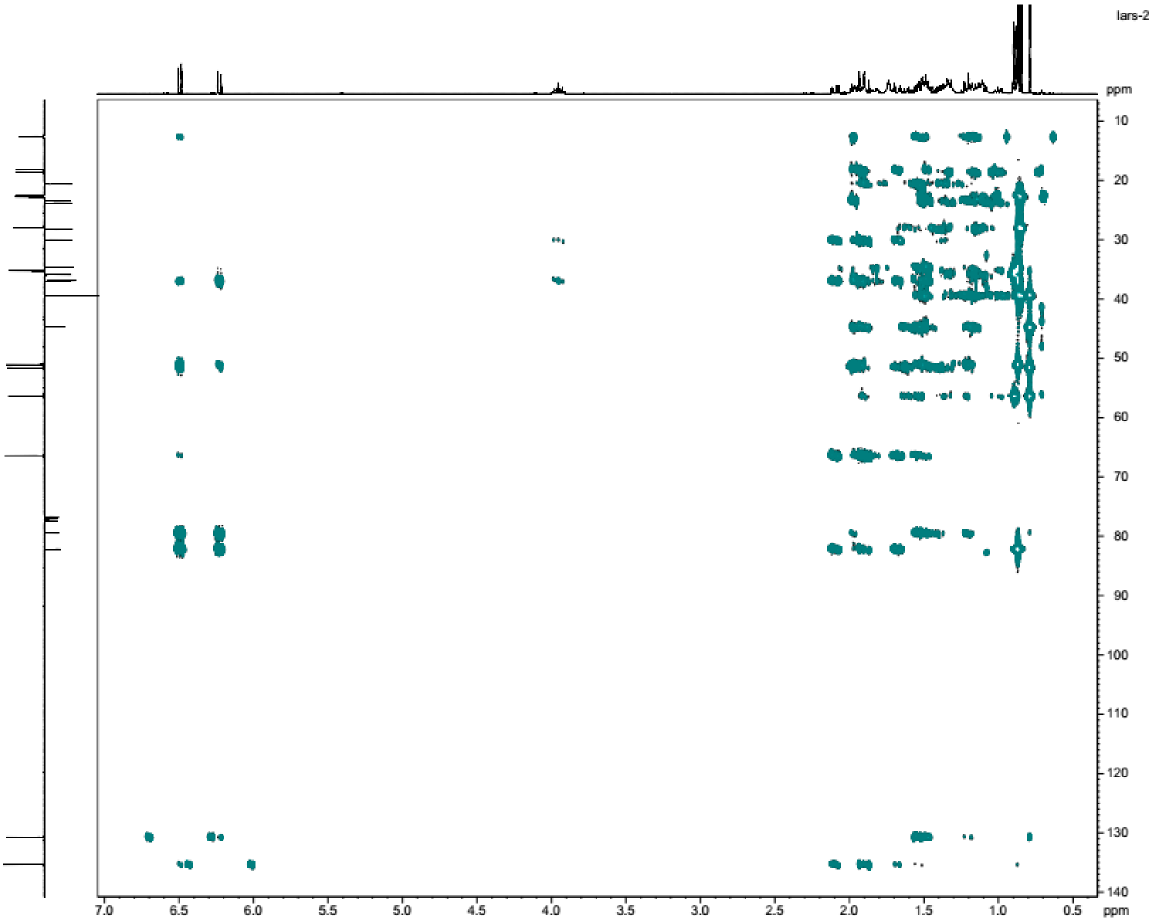

Figure S7. gs-HMBC of DHCholEP.

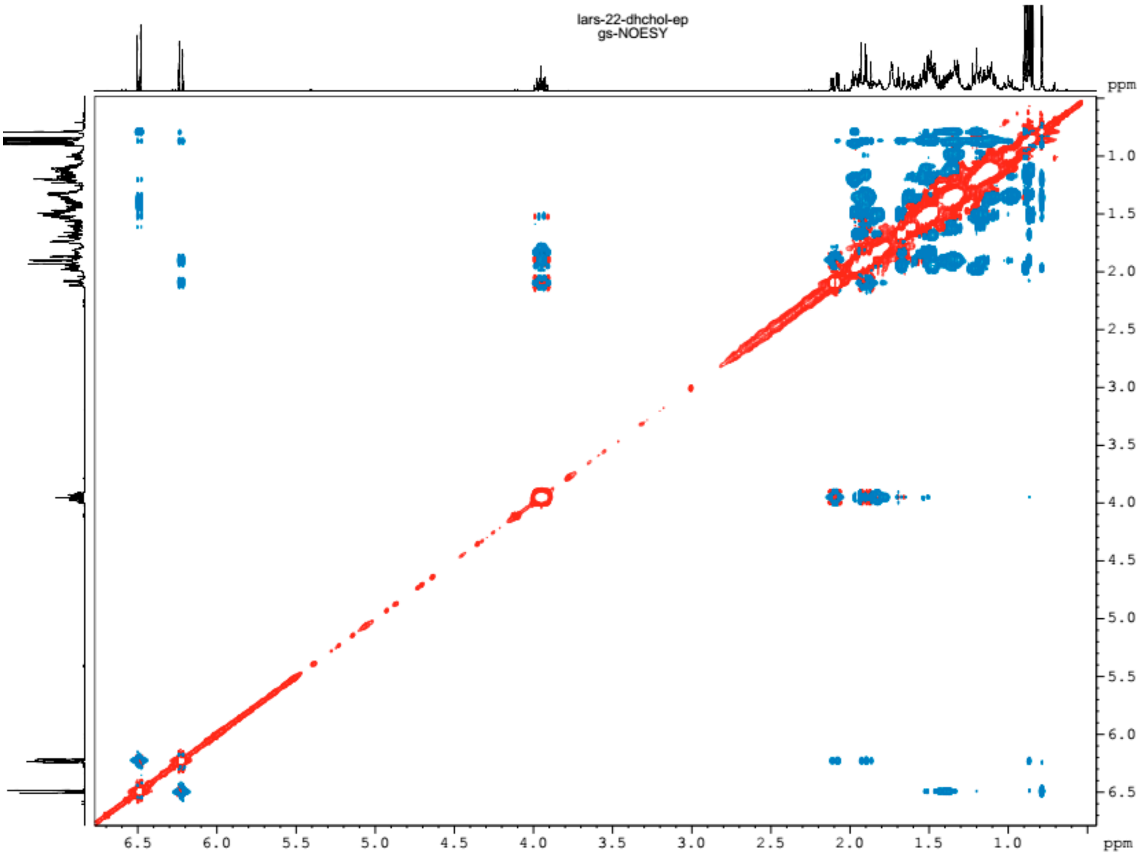

Figure S8. gs-NOESY of DHCholEP.

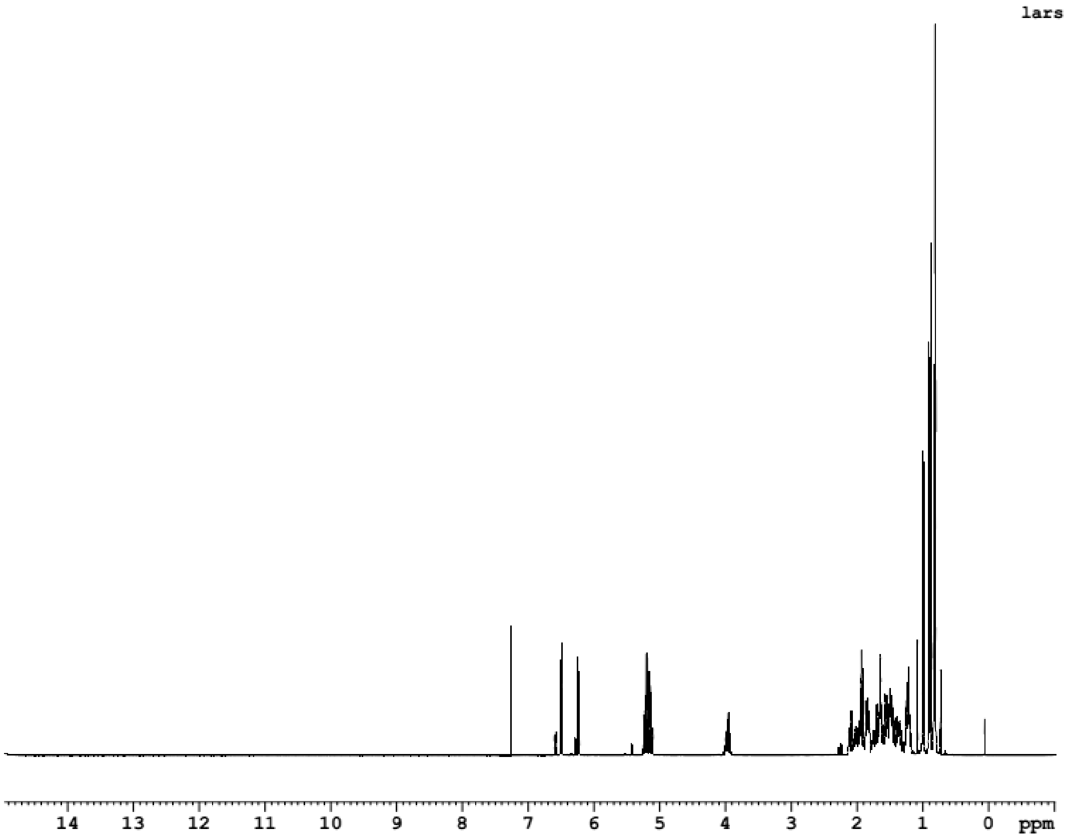

Figure S9. ^{1}H NMR of mixture of ErgoEP and DHErgolEP.

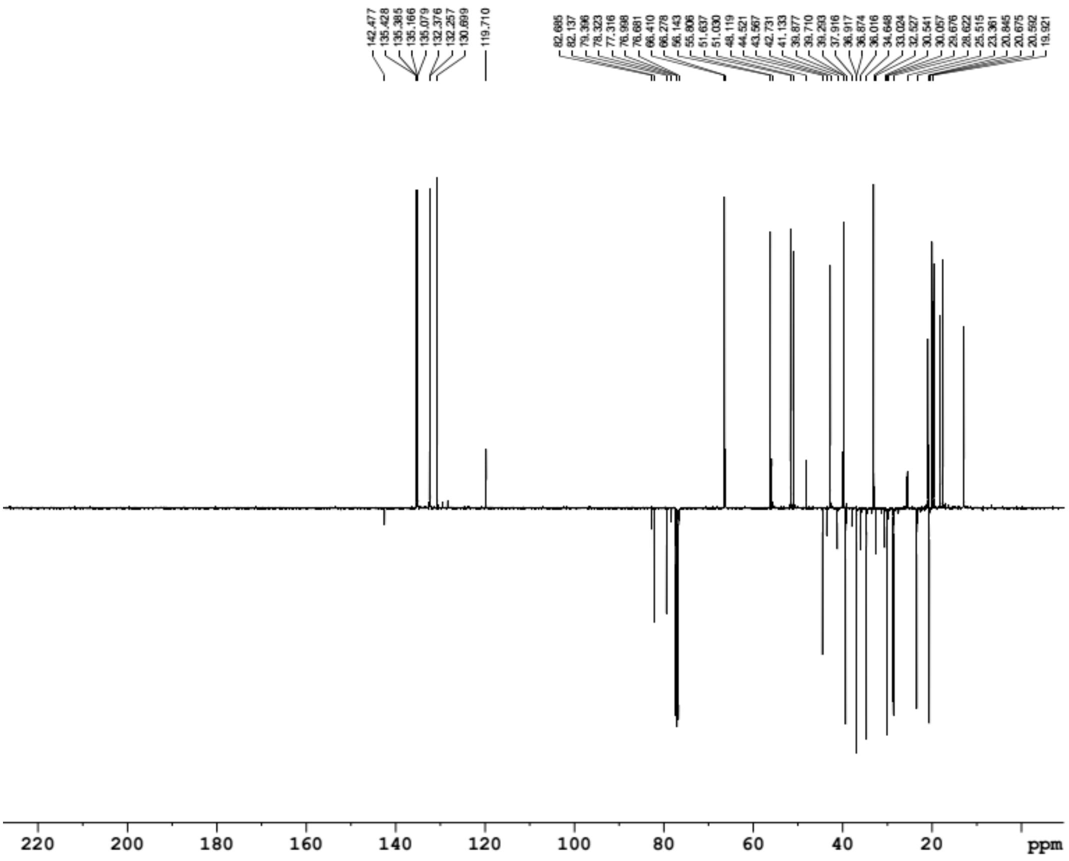

Figure S10. ^{13}C NMR of mixture of ErgoEP and DHErgolEP.

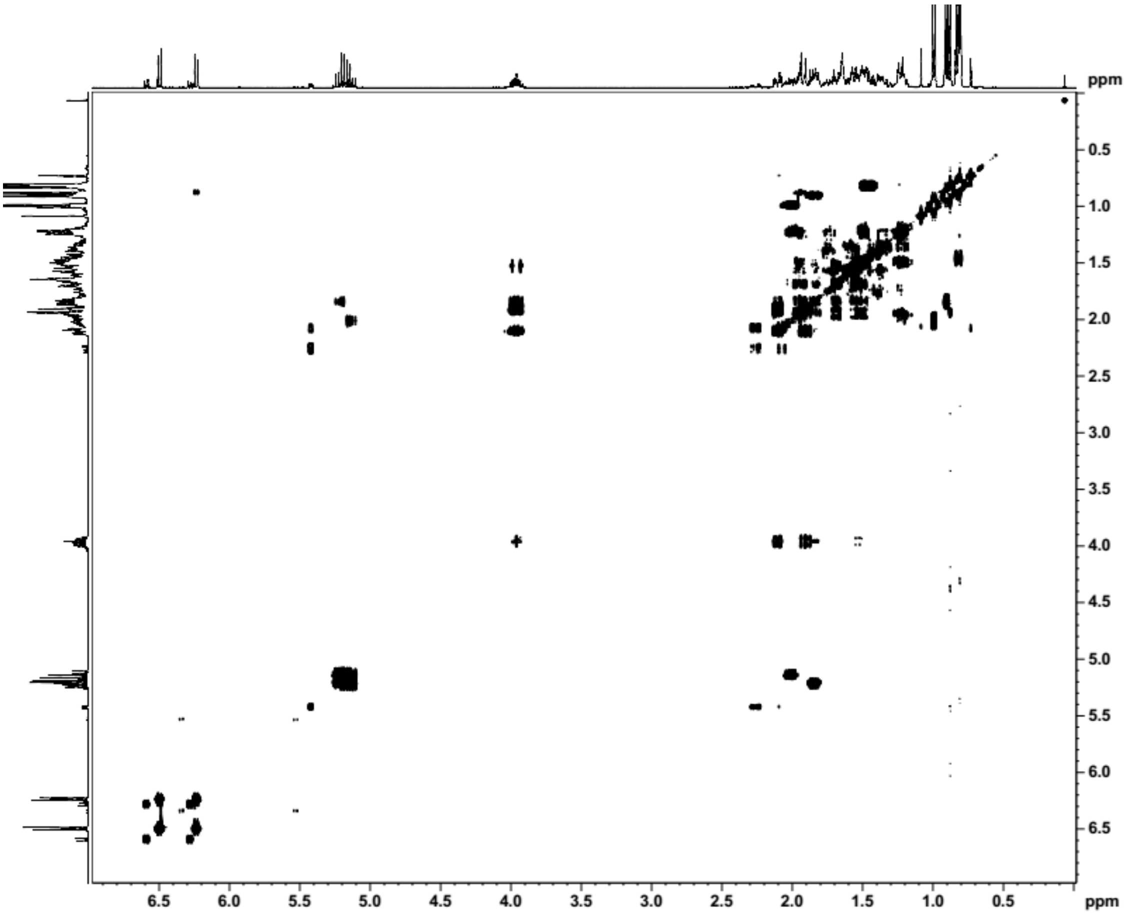

Figure S11. COSY of mixture of ErgoEP and DHErgolEP.

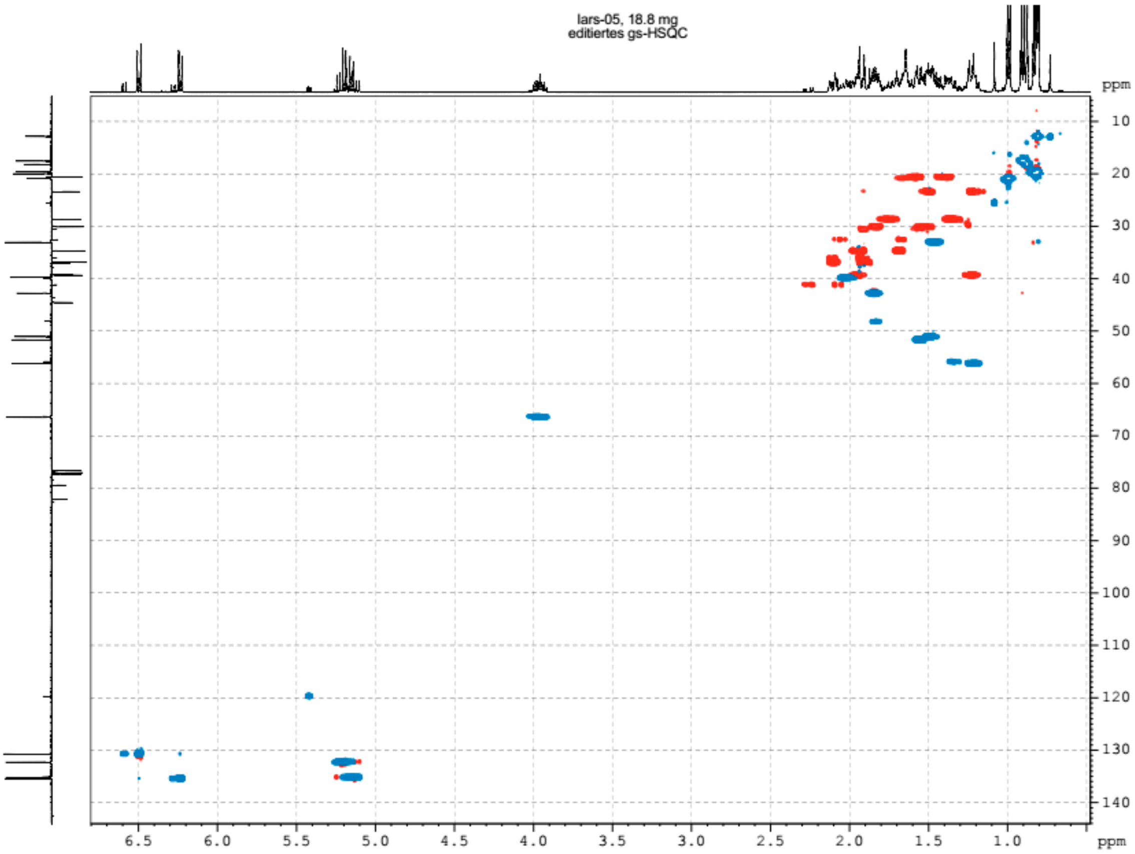

Figure S12. edited gs-HSQC of mixture of ErgoEP and DHErgolEP.

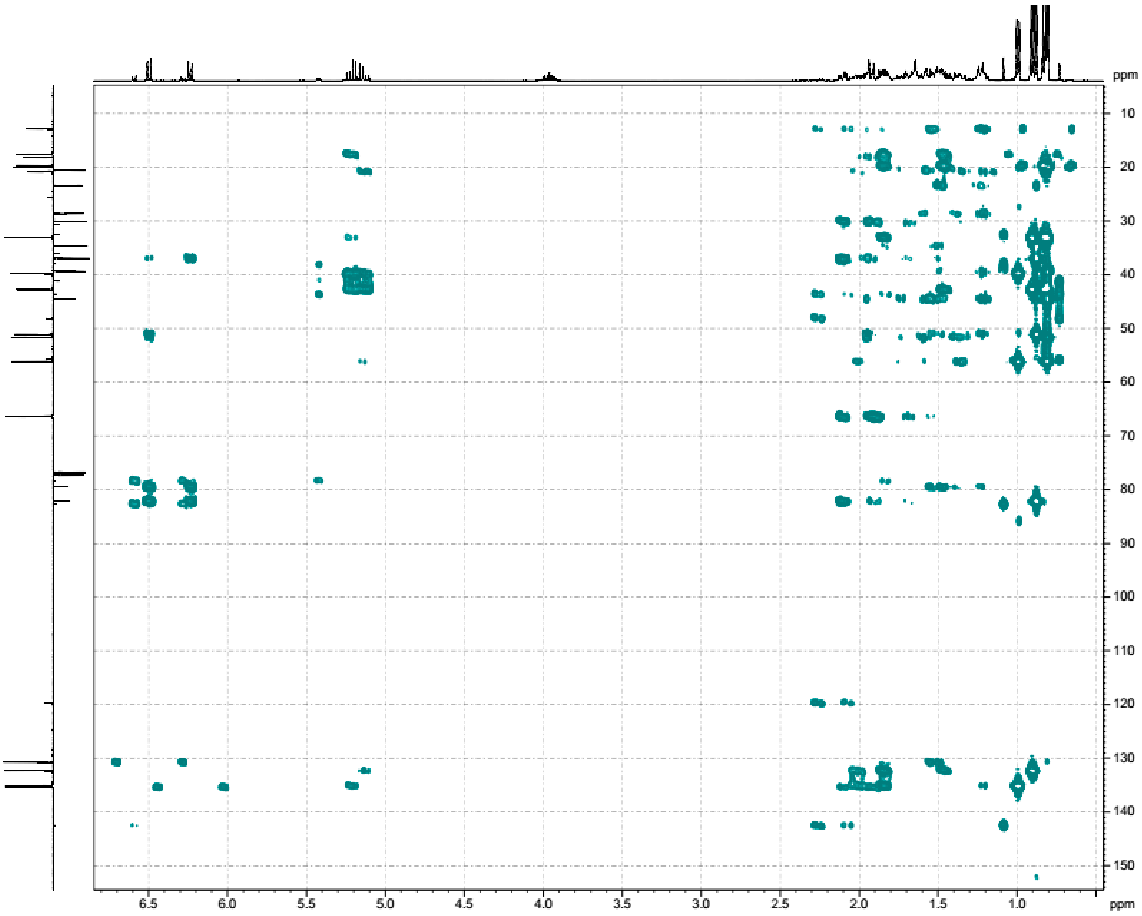

Figure S13. gs-HMBC of mixture of ErgoEP and DHErgolEP.

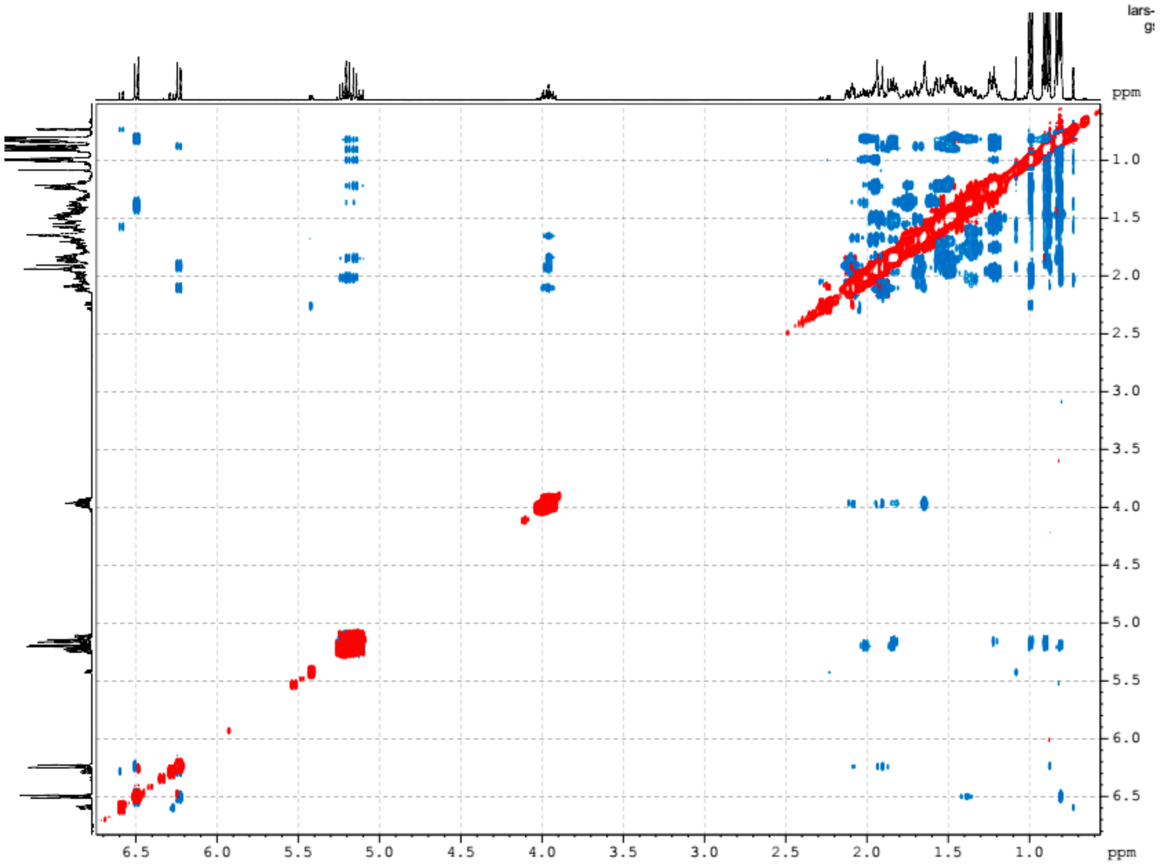

Figure S14. gs-NOESY of mixture of ErgoEP and DHErgolEP.
.

Table S4. Extended parameters used for the simulation in the program WINSIM 2002. EPR spectra were obtained during the reaction of sterols and sterol EPs with Fe^{2+} using DMPO as spin trap compound. Simulated spectral parameters and tentative assignments are given. Below each compound the contribution of different spin adducts with their nitrogen- (a_N) and hydrogen- ($a_{ßH}$, $a_{\gamma H}$) coupling constants and their ratio (a_N/a_H) are listed.

Compound, Species No.	Correlation Coefficient	Spin Adduct Contrib. (%)	Line Shape Lorentzian (%)	Line Width (G)	g at H0 (g)	g-Shift (G)	a_N (G)	$a_{ßH}$ (G)	$a_{\gamma H}$ (G)	a_N / a_H	Assignment
ErgoEP	0.964				2.0062						
1		62.951	100.0	1.270		0.248	14.200	9.520	1.550	1.49	RO•
2		19.510	60.0	0.797		0.433	14.771	18.177	-	0.81	-
3		17.539	50.0	1.047		0.475	15.533	22.713	-	0.68	RC•
DHCholEP	0.874				2.0063						
1		38.250	5.0	1.032		0.675	15.995	22.083	-	0.72	RC•
2		27.651	50.4	0.788		0.578	16.503	14.804	-	1.11	ROO•
3		21.303	12.7	0.686		0.546	13.900	9.641	1.393	1.45	RO•
4		12.796	0.0	0.689		0.553	14.974	12.132	-	1.23	ROO•
Ergo	0.945				2.0058						
1		45.310	100.0	1.096		-0.390	14.018	9.366	1.496	1.48	RO•
2		36.052	100.0	1.066		-0.297	14.6	13.5	-	1.08	ROO•
3		16.954	100	1..323		-0.098	15.520	22.948	-	0.67	RC•
4		1.685	97.9	0.351		-0.293	16.305	14.940	-	1.09	ROO•
DHChol	0.961				2.0058						
1		63.745	100.0	0.990		-0.193	14.562	13.550	-	1.07	ROO•
2		23.728	100.0	1.065		0.0	13.809	9.126	1.349	1.51	RO•
3		10.250	64.2	0.911		0.0	15.486	22.566	-	0.68	RC•
4		2.276	100.0	0.522		0.0	14.883	18.054	-	0.83	-

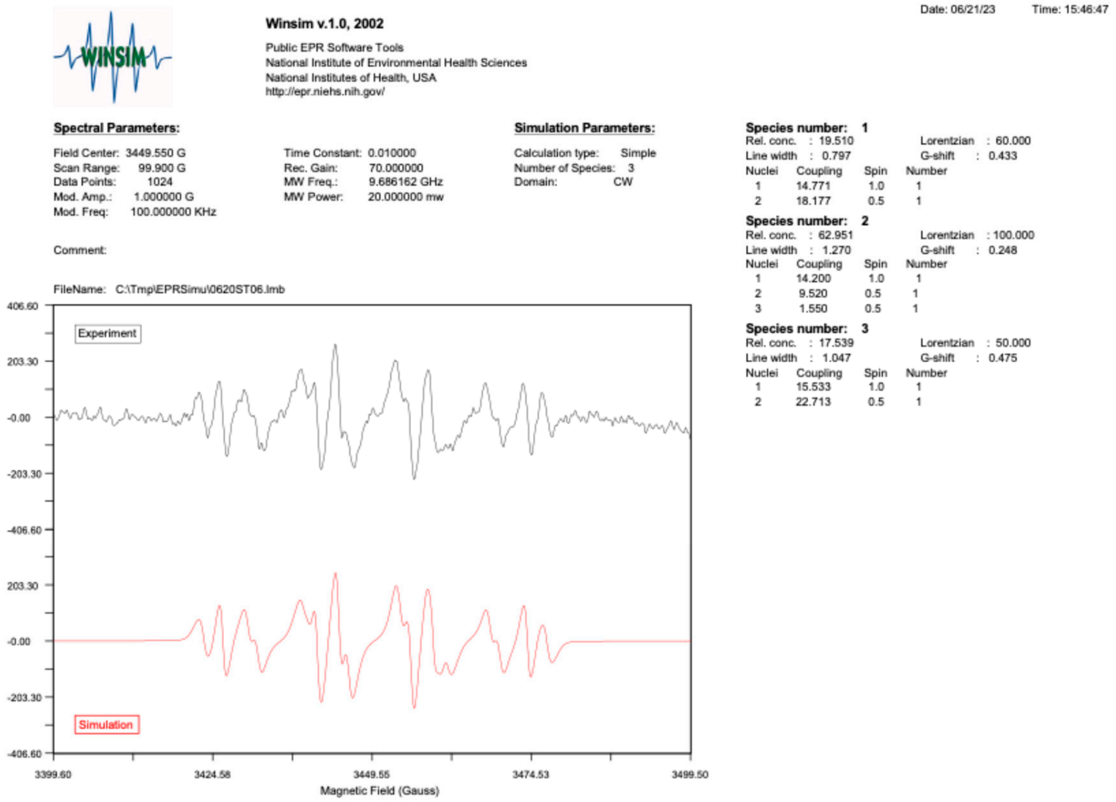

Figure S15. Simulation of DMPO spin adducts obtained from the reaction of ErgoEP with Fe^{2+}.

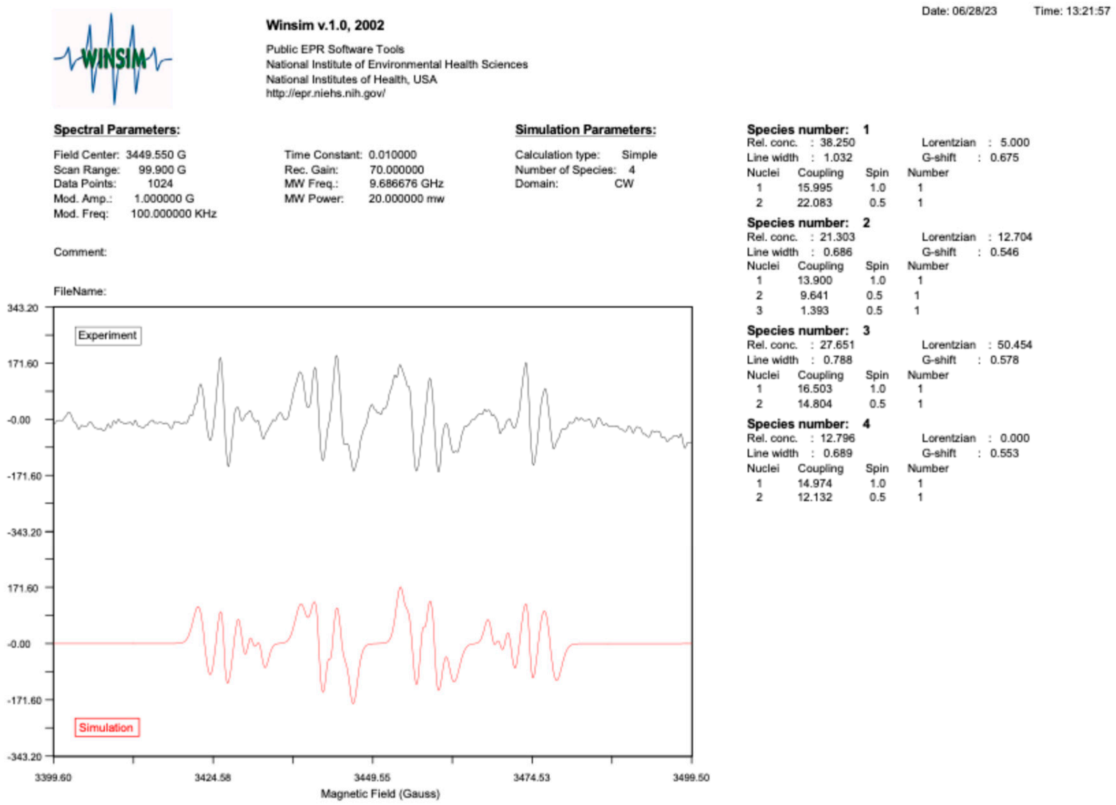

Figure S16. Simulation of DMPO spin adducts obtained from the reaction of DHCholEP with Fe^{2+}.

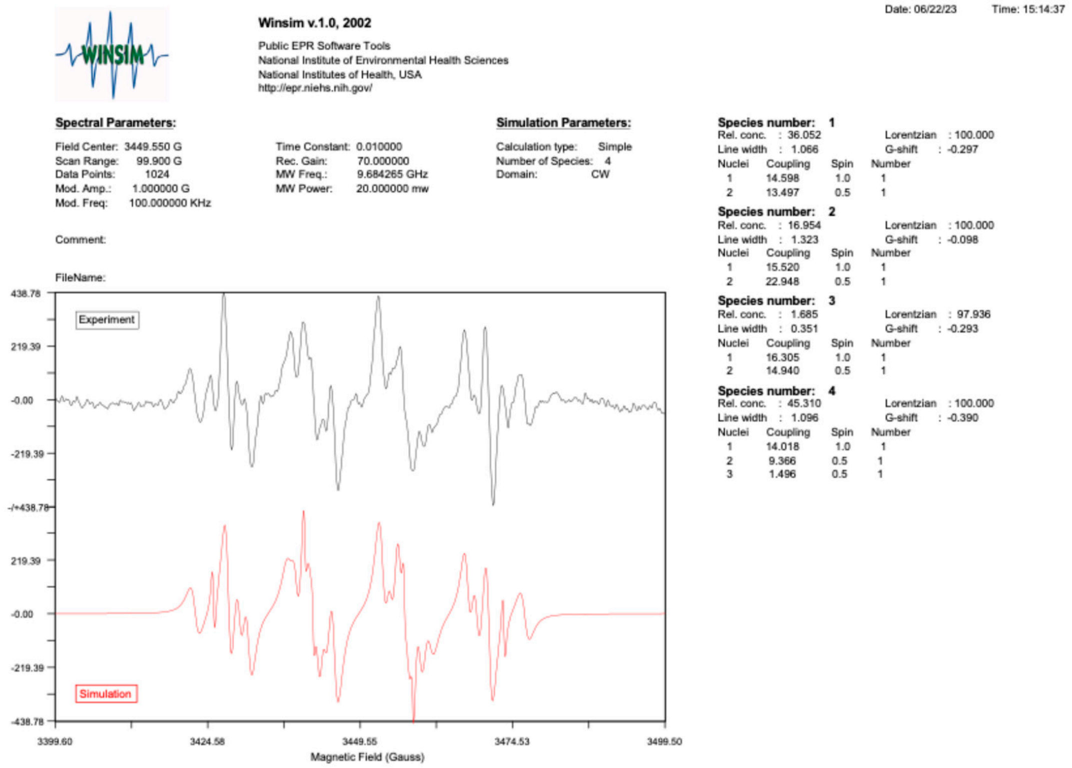

Figure S17. Simulation of DMPO spin adducts obtained from the reaction of Ergo with Fe^{2+}.

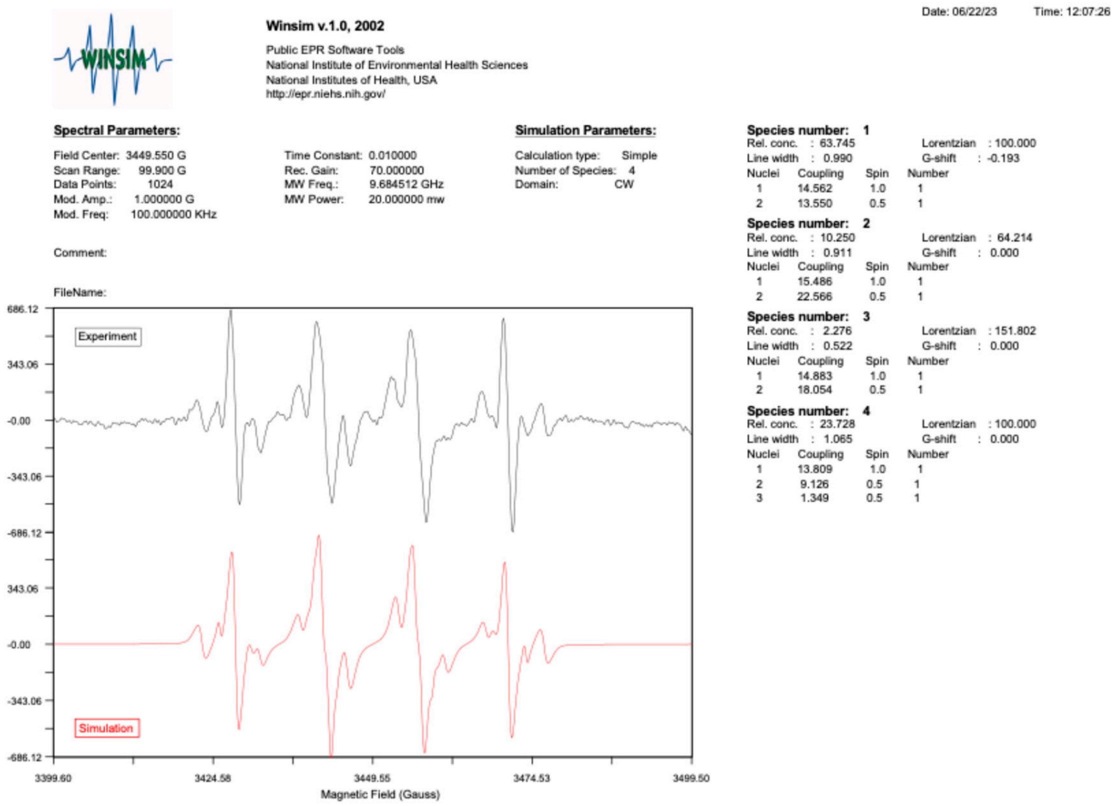

Figure S18. Simulation of DMPO spin adducts obtained from the reaction of DHChol with Fe^{2+}.
